# Multifunctional bimetallic Ag–CuO and Ag–ZnO nanoparticles biosynthesized using *Plantago ovata* for biomedical applications

**DOI:** 10.1039/d6ra05100f

**Published:** 2026-09-25

**Authors:** Hayfa Habes Almutairi, Nada H. Aljarba, Sahar M. AlMotwaa, Waad A. Al-Otaibi, Mohamed K. Y. Soliman

**Affiliations:** a Chemistry Department, College of Science, King Faisal University Al-Ahsa Saudi Arabia Halmutairi@kfu.edu.sa; b Department of Biology, College of Science, Princess Nourah Bint Abdulrahman University P.O. Box 84428 Riyadh 11671 Saudi Arabia nhaljarba@pnu.edu.sa; c Applied College, Shaqra University Shaqra 11961 Saudi Arabia saharm@su.edu.sa; d Department of Chemistry, College of Science and Humanities, Shaqra University Shaqra 11961 Saudi Arabia w.otaibi@su.edu.sa; e Botany and Microbiology Department, Faculty of Science, Al-Azhar University Nasr City 11884 Cairo Egypt Mohamed.k.yousef@azhar.edu.eg Halmutairi@kfu.edu.sa

## Abstract

This study investigated the green synthesis, physicochemical characteristics, and biological activities of Ag–CuO and Ag–ZnO mixed-phase nanomaterials using *Plantago ovata* seed extract. Monometallic AgNPs, CuONPs, and ZnONPs, the free extract, and metal-salt precursors were included as comparators. Three independently prepared extract batches showed limited batch-to-batch variability in pH, dry-solids concentration, and extraction yield, with coefficient-of-variation values below 9%. UV-vis, FTIR, XRD, HR-TEM, EDX, and elemental mapping collectively supported the coexistence of Ag and the corresponding CuO or ZnO crystalline phases. TEM-derived mean particle sizes were 72.02 ± 28.49 and 83.30 ± 32.10 nm for Ag–CuONPs and Ag–ZnONPs, respectively, while their Z-average hydrodynamic diameters were 103.8 and 124.4 nm. Their zeta potentials were −36.1 and −28.1 mV, respectively. Both mixed-phase formulations exhibited broad-spectrum antibacterial and concentration-dependent antibiofilm activities. Ag–CuONPs generally showed stronger broth-dilution activity, with minimum inhibitory concentrations of 125–500 µg mL^−1^, and produced maximum biofilm inhibition of 84.91% against *Staphylococcus aureus* and 61.78% against *Pseudomonas aeruginosa*. Both formulations increased extracellular protein leakage and reduced the expression of biofilm- and virulence-associated genes, including *fnbA*, *cna*, *algD*, and *lasB*. In cancer-cell assays, Ag–CuONPs showed the greatest activity against Caco-2 cells, with an IC_50_ of 102.89 ± 8.05 µg mL^−1^, whereas Ag–ZnONPs were most active against MCF-7 cells, with an IC_50_ of 116.98 ± 3.23 µg mL^−1^. Annexin V/PI analysis confirmed substantial apoptosis in Caco-2 cells, while Ag–ZnONPs induced pronounced G_2_/M-phase accumulation. Both treatments increased *BAX* and *CASP3* expression and decreased *BCL2* expression. Ag–CuONPs exhibited the strongest DPPH-scavenging activity among the nanomaterials, whereas Ag–ZnONPs showed the greatest ABTS-scavenging activity. Overall, the results demonstrate composition-dependent antibacterial, antibiofilm, radical-scavenging, and anticancer activities of the *P. ovata*-mediated mixed-phase nanomaterials, supporting their potential for further biomedical investigation while requiring additional mechanistic and *in vivo* validation.

## Abstract 2

This study investigated the green synthesis, physicochemical characteristics, and biological activities of Ag–CuO and Ag–ZnO mixed-phase nanomaterials using *Plantago ovata* seed extract.

## Graphical abstract

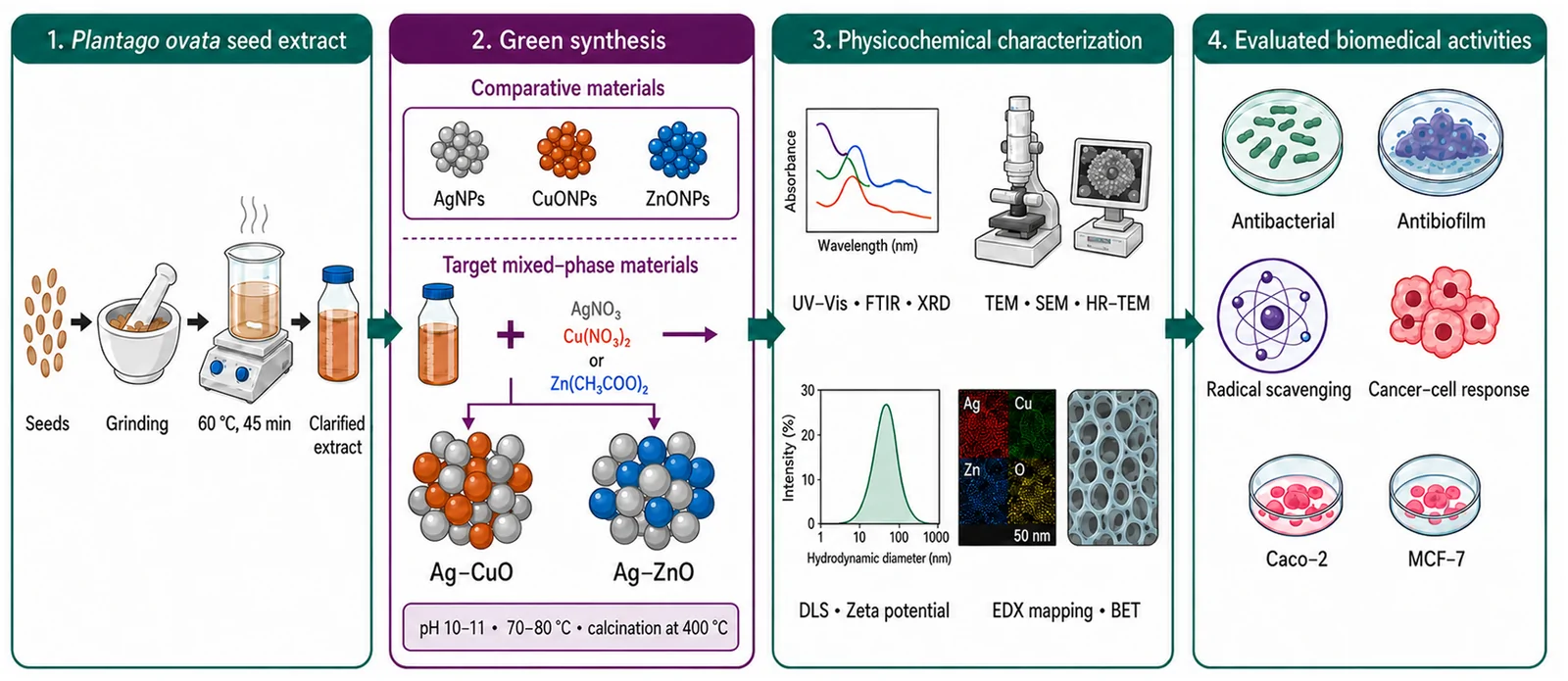

## 1 Introduction

Cancer remains a leading cause of morbidity and mortality worldwide and continues to impose a substantial clinical and socioeconomic burden owing to its increasing incidence, biological complexity, and high treatment costs.^1^ Tumour heterogeneity, genetic instability, and the persistence of cancer stem-cell populations can promote disease progression, therapeutic resistance, recurrence, and metastasis.^2^ Although chemotherapy remains an essential component of cancer treatment, its clinical effectiveness may be limited by acquired or intrinsic resistance and by treatment-associated toxicities affecting both malignant and healthy tissues.^3,4^ In parallel with cancer, antimicrobial resistance represents another major global health challenge. Infections caused by multidrug-resistant microorganisms are associated with increased morbidity, mortality, hospitalization, and healthcare expenditure, particularly among immunocompromised patients.^5,6^ Biofilm formation further complicates the treatment of bacterial infections by generating an extracellular matrix that promotes surface attachment, restricts antimicrobial penetration, alters cellular metabolism, and facilitates the persistence of tolerant bacterial populations.^7^

Nanomaterials have emerged as promising candidates for biomedical applications because their physicochemical and biological behaviour can be influenced by composition, particle size, morphology, crystallinity, aggregation state, surface charge, and surrounding medium. These characteristics can increase particle–cell interactions and generate biological responses that differ from those of the corresponding bulk materials.^8^ However, conventional physical and chemical synthesis methods may require high energy consumption, specialized equipment, or hazardous reducing and stabilizing chemicals, potentially limiting their environmental sustainability.^9^ Green-synthesis strategies employing plants, bacteria, fungi, or algae provide an alternative route in which biological constituents may participate in precursor coordination, reduction, nucleation, particle growth, and surface stabilization.^10^ Among the nanomaterials investigated for biomedical purposes, metallic Ag nanoparticles and the semiconducting metal-oxide nanoparticles CuO and ZnO have received considerable attention. CuO and ZnO nanoparticles possess composition- and size-dependent optical, electronic, catalytic, and surface properties that support their investigation in antimicrobial, anticancer, sensing, and environmental applications.^11^ AgNPs are also well recognized for their broad-spectrum antimicrobial activity and their capacity to interact with microbial membranes and intracellular components.^12^ Nevertheless, the biological behavior of these materials varies substantially with synthesis conditions, surface chemistry, aggregation, exposure concentration, and the tested biological model. Their performance and safety therefore require direct experimental evaluation rather than assumptions based solely on nominal composition.^13^ Combining Ag with CuO or ZnO offers an approach for modifying the structural, optical, surface, and biological characteristics of the corresponding individual components. Ag-containing CuO materials have been reported to show altered crystallite dimensions, optical responses, and antimicrobial performance relative to undoped or unmodified CuO.^14^ Plant-assisted synthesis has also enabled the preparation of crystalline Ag–CuO materials with composition-dependent physicochemical properties.^15^ Similarly, green-synthesized Ag–ZnO nanomaterials have shown the coexistence of Ag- and ZnO-related phases and, in some systems, improved antimicrobial performance relative to monometallic ZnO.^16,17^

*Plantago ovata* Forssk., belonging to the family Plantaginaceae and commonly known as psyllium or Qatuna, is an economically and medicinally important plant cultivated particularly in India, Pakistan, Iran, and other arid or semiarid regions. Its seeds possess a mucilage-rich outer layer with strong hydration, swelling, viscosity, and gel-forming properties. Psyllium has been investigated for its beneficial effects on gastrointestinal function, glycaemic regulation, serum lipids, body weight management, and cardiovascular risk factors.^18^ In addition to its fibre-rich mucilage, *P. ovata* seeds and husks have been reported to contain phenolic acids, flavonoids, and other water-extractable constituents with hydroxyl and carbonyl-containing functional groups.^19^ Such constituents may provide a suitable chemical environment for precursor coordination and particle formation during aqueous green synthesis. Based on the HPLC profile obtained in the present study, *P. ovata* was selected because its aqueous seed extract contained cinnamic acid, apigenin, and other water-extractable constituents bearing functional groups that may participate in precursor coordination, reduction, nucleation, and stabilization during green synthesis (Table 1). Although the free extract possesses intrinsic biological activity, its primary role in this study was to provide a plant-derived synthesis medium. Combining Ag with CuO or ZnO was intended to produce mixed-phase materials with physicochemical and biological properties distinct from those of the free extract and corresponding monometallic nanoparticles. The comparative design therefore evaluated whether the final Ag–CuO and Ag–ZnO formulations provided broader functional performance without presuming synergism.

**Table 1:** HPLC quantitative profiling of phenolic and flavonoid compounds in *Plantago ovata* (Qatuna) seeds

No.	Peak name	Retention time (min)	Area (mAU·min^−1^)	Height (mAU)	Relative area (%)	Relative height (%)	Amount (µg g^−1^)
1	Apigenin	1.533	101.857	1021.080	34.00	73.59	5086.82
2	Diosmin	4.233	1.803	11.503	0.60	0.83	0.14
3	Rutin	5.533	6.231	23.826	2.08	1.72	4.03
4	Hesperidin	7.207	69.833	138.766	23.31	10.00	154.43
5	Kaempferol	12.467	72.369	130.094	24.16	9.38	60.13
6	Quercetin	13.637	47.468	62.227	15.85	4.48	149.21
7	Quinic acid	2.343	22.222	234.088	2.86	19.65	593.45
8	Gallic acid	2.550	1.240	19.578	0.16	1.64	24.25
9	Chlorogenic acid	4.023	0.146	3.653	0.02	0.31	5.55
10	Resorcinol	4.540	0.166	3.834	0.02	0.32	8.28
11	Pyrocatechol	5.260	0.077	2.908	0.01	0.24	10.30
12	Vanillic acid	6.147	16.781	32.561	2.16	2.73	117.10
13	Coumaric acid	7.803	16.336	42.487	2.10	3.57	74.28
14	Ellagic acid	9.160	0.270	7.408	0.03	0.62	0.90
15	Ferulic acid	9.730	3.485	10.084	0.45	0.85	36.00
16	Cinnamic acid	13.730	717.090	834.696	92.19	70.07	6385.35

Accordingly, this study hypothesizes that the aqueous extract of *Plantago ovata* seeds can mediate the green formation of Ag–CuO and Ag–ZnO mixed-phase nanomaterials and that combining Ag with CuO or ZnO may produce biological responses distinct from those of the corresponding individual components. *P. ovata* was selected because HPLC profiling identified cinnamic acid and apigenin, whose functional groups may contribute to precursor coordination, nucleation, reduction, and stabilization during nanomaterial formation. Here, “multifunctional” refers specifically to the experimentally evaluated antibacterial, antibiofilm, radical-scavenging, and anticancer-related cellular activities of the final nanomaterials, whereas coordination, reduction, nucleation, and stabilization describe the proposed roles of the extract during synthesis. These proposed roles do not establish that an intact phytochemical coating remained on the calcined materials. To distinguish the contributions of the different components, Ag–CuO and Ag–ZnO were compared with monometallic AgNPs, CuONPs, and ZnONPs, free plant extract, and the corresponding metal-salt precursors. This comparative design evaluates composition-dependent functional differences without presuming synergism. The study also examines cellular responses in Caco-2 and MCF-7 cancer cells relative to non-malignant Vero cells and investigates their association with apoptosis, cell-cycle alterations, and changes in *CASP3*, *BAX*, and *BCL2* expression.

## 2 Materials and methods

### 2.1 Materials

All chemicals, reagents, and biological materials used in this study are listed in Table S1 (SI).

### 2.2 High-performance liquid chromatography (HPLC)

A representative *Plantago ovata* seed extract prepared under the standardized extraction conditions described above was subjected to phytochemical profiling using an Agilent 1260 Infinity II HPLC system equipped with a diode-array detector (DAD). Chromatographic separation was performed on an Eclipse XDB-C18 column using a gradient mobile phase composed of acetonitrile and 2% (v/v) aqueous acetic acid at a flow rate of 0.8 mL min^−1^. Before analysis, the extract was filtered through a 0.45-µm membrane filter. Detection was performed at 280, 320, and 360 nm. Compounds were identified by comparing their retention times and UV spectra with those of authentic standards. This analysis was used to characterize the representative phytochemical profile of the extract and was not included in the assessment of batch-to-batch reproducibility.

### 2.3 Preparation and batch standardization of *Plantago ovata* extract

To evaluate the reproducibility of the extraction procedure, three independent *Plantago ovata* aqueous extract batches (E1–E3) were prepared separately from the same homogenized seed lot under identical conditions. For each batch, 5.0 g of finely ground seeds was extracted with 100 mL of distilled water at 60 °C for 45 min under continuous stirring. After cooling to room temperature, the extract was centrifuged at 5000 rpm for 10 min, filtered through Whatman No. 1 filter paper, and adjusted to a final volume of 100 mL with distilled water. The pH of each batch was recorded. Dry-solids concentration was determined gravimetrically by drying three separate 5-mL aliquots from each batch at 60 °C until a constant mass was obtained. Dry-solids concentration and extraction yield were calculated using the following equations:Dry-solids concentration (mg mL^−1^) = dried-residue mass (mg)/aliquot volume (mL)Extraction yield (%) = [dry-solids concentration (mg mL^−1^) × final extract volume (mL)/initial dry seed mass (mg)] × 100

Extraction reproducibility was evaluated by comparing the pH, dry-solids concentration, and extraction yield of E1–E3. The results were expressed as mean ± standard deviation (SD), and the coefficient of variation was calculated as CV (%) = (SD/mean) × 100.

### 2.4 Green synthesis of monometallic and bimetallic nanomaterials

**Fig. 1:**
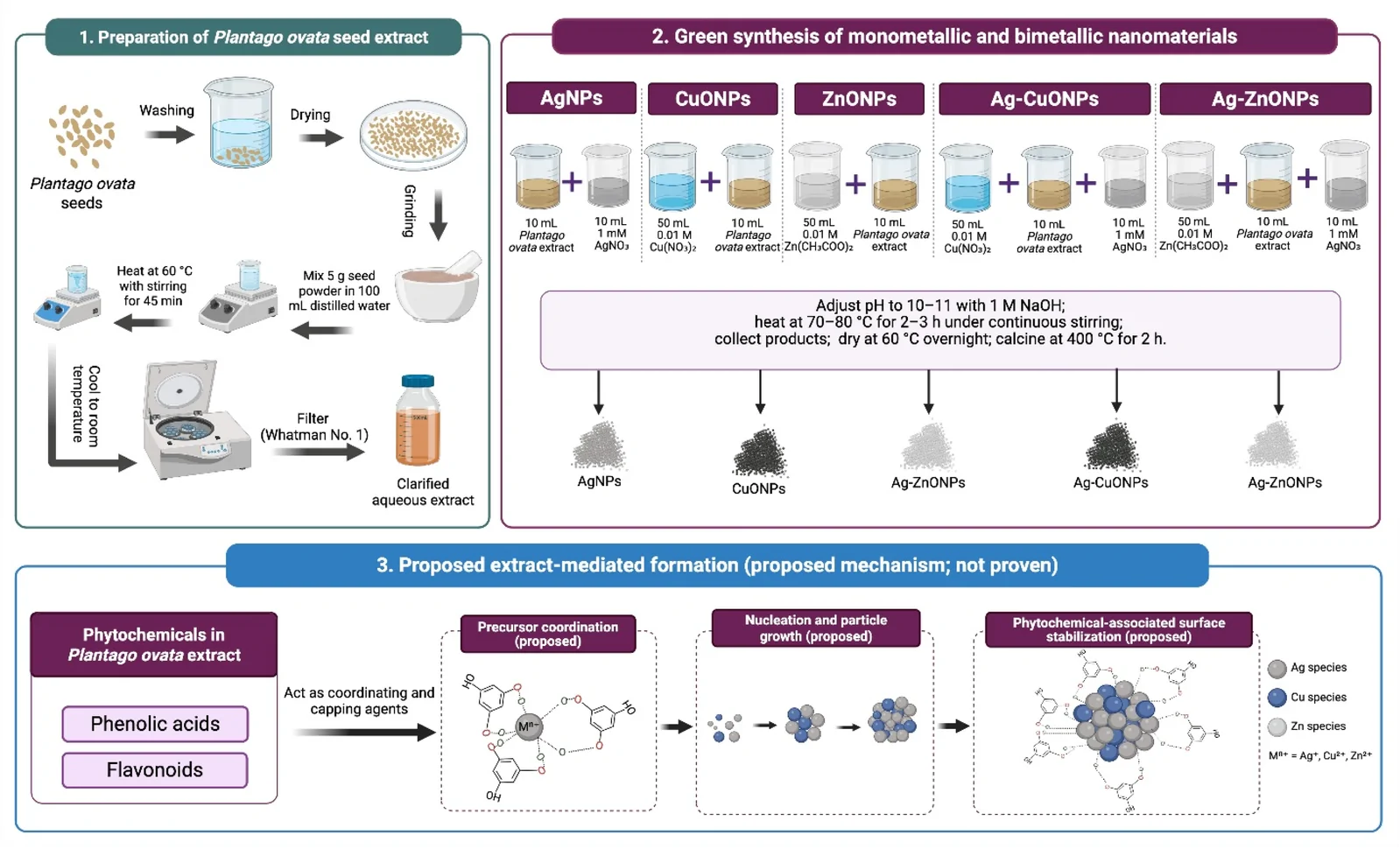
Schematic representation of the preparation of *Plantago ovata* seed extract, green synthesis of monometallic AgNPs, CuONPs, and ZnONPs and bimetallic Ag–CuONPs and Ag–ZnONPs, and the proposed extract-mediated formation pathway involving precursor coordination, nucleation, particle growth, and phytochemical-associated surface stabilization. The mechanistic pathway is proposed based on the experimentally identified phenolic acids and flavonoids and supporting literature and should not be interpreted as direct mechanistic evidence.

Monometallic AgNPs, CuONPs, and ZnONPs and bimetallic Ag–CuONPs and Ag–ZnONPs were synthesized using *Plantago ovata* seed extract as a plant-derived reducing and stabilizing medium. The phytochemical constituents of the extract were expected to participate in precursor coordination, nucleation, particle growth, and surface stabilization during nanomaterial formation. For Ag–CuONPs synthesis, 50 mL of 0.01 M copper nitrate solution was mixed with 10 mL of *P. ovata* seed extract under continuous stirring. Subsequently, 10 mL of 1 mM AgNO_3_ solution was added gradually to the reaction mixture. The pH was adjusted to 10–11 using 1 M NaOH, and the mixture was heated at 70–80 °C for 2–3 h until a dark precipitate was formed. Ag–ZnONPs were prepared using the same procedure, with zinc nitrate replacing copper nitrate. Briefly, 50 mL of 0.01 M Zn(NO_3_)_2_ solution was mixed with 10 mL of the plant extract, followed by the gradual addition of 10 mL of 1 mM AgNO_3_ solution. The pH was adjusted to 10–11 using 1 M NaOH, and the reaction mixture was maintained at 70–80 °C for 2–3 h until a white-to-pale-brown precipitate was obtained. The corresponding monometallic nanomaterials were synthesized separately for comparative physicochemical characterization and biological evaluation. AgNPs were prepared by mixing 10 mL of 1 mM AgNO_3_ solution with 10 mL of *P. ovata* seed extract under continuous stirring. CuONPs were prepared by mixing 50 mL of 0.01 M copper nitrate solution with 10 mL of the plant extract, whereas ZnONPs were synthesized by mixing 50 mL of 0.01 M Zn(NO_3_)_2_ solution with 10 mL of the extract. For each monometallic formulation, the pH was adjusted to 10–11 using 1 M NaOH, and the reaction mixture was heated at 70–80 °C for 2–3 h to promote nanomaterial formation. The resulting products were collected and dried at 60 °C overnight. The dried materials were subsequently calcined at 400 °C for 2 h to obtain crystalline AgNPs, CuONPs, ZnONPs, Ag–CuONPs, and Ag–ZnONPs. The synthesized monometallic nanomaterials were used as comparator groups to determine whether the physicochemical and biological properties of the Ag–CuO and Ag–ZnO formulations differed from those of their corresponding individual components. The overall extract-mediated synthesis process is illustrated in Fig. 1.

### 2.5 Characterization study of nanoparticles

The physicochemical properties of the biosynthesized monometallic AgNPs, CuONPs, and ZnONPs and bimetallic Ag–CuONPs and Ag–ZnONPs were evaluated using complementary analytical techniques. UV-visible absorption spectra were recorded over the wavelength range of 200–800 nm using a JENWAY 6305 UV-visible spectrophotometer (UK) to examine the optical characteristics of the synthesized materials. Crystalline structures and phase compositions were investigated by X-ray diffraction using a Philips X'Pert Pro diffractometer equipped with Cu Kα radiation over a 2*θ* range of 10°–80°. Fourier-transform infrared spectra were recorded over the range of 400–4000 cm^−1^ using a JASCO FT-IR 3600 spectrometer to identify surface-associated functional groups and metal–oxygen-related vibrational bands. The hydrodynamic diameter and polydispersity index of the nanomaterial suspensions were determined by dynamic light scattering using a Zetasizer Nano ZS instrument (Malvern Instruments, UK), while their surface charge was evaluated by zeta-potential measurements using a NICOMP™ 380 ZLS analyzer. Particle morphology, aggregation state, and dry-state particle-size distributions were examined by scanning electron microscopy using a ZEISS EVO-MA10 microscope (Germany) and transmission electron microscopy using a JEM-2100Plus microscope (JEOL Ltd, Japan). High-resolution transmission electron microscopy was additionally performed using the JEM-2100Plus for the bimetallic Ag–CuONPs and Ag–ZnONPs to visualize their lattice fringes and provide further evidence for the coexistence of the corresponding crystalline phases. Elemental composition and spatial elemental distribution were evaluated using energy-dispersive X-ray spectroscopy and elemental mapping with a Bruker detector. The surface area and pore characteristics of Ag–CuONPs and Ag–ZnONPs were evaluated nitrogen adsorption–desorption analysis using a Belsorp-mini X analyzer (MicrotracBEL, Japan). The specific surface area was calculated using the Brunauer–Emmett–Teller method, whereas pore-size distribution and pore volume were estimated using the Barrett–Joyner–Halenda method.

### 2.6 Antibacterial and antibiofilm activities of Ag–CuONPs and Ag–ZnONPs

#### 2.6.1 Antibacterial susceptibility and MIC/MBC determination

The antibacterial activities of the monometallic AgNPs, CuONPs, and ZnONPs and bimetallic Ag–CuONPs and Ag–ZnONPs were evaluated against five reference bacterial strains: *Klebsiella pneumoniae* ATCC 13883, *Pseudomonas aeruginosa* ATCC 9027, *Bacillus subtilis* ATCC 6633, *Staphylococcus aureus* ATCC 35556, and *Escherichia coli* ATCC 8739. The antibacterial activities of *Plantago ovata* extract and the corresponding metal precursors, AgNO_3_, Cu(NO_3_)_2_, and Zn(NO_3_)_2_, were also examined as comparator groups to distinguish the effects of the synthesized nanomaterials from those of the free extract and metal salts. Antibacterial susceptibility was initially assessed using the agar well-diffusion assay.^20^ Briefly, standardized strains inoculum was swabbed onto Mueller–Hinton agar (MHA) plates, into which 6-mm holes were aseptically punched and introduced by about 100 µL of tested nanoparticle suspension (1000 µg mL^−1^). Following a 24-h in incubator at 37 °C, the distance of the resulting clear area around the well was measured in triplicate. The MIC values were determined using the broth microdilution method in 96-well plates according to CLSI guidelines.^21^ The nanomaterial suspensions were subjected to two-fold serial dilution in Mueller–Hinton broth to obtain final concentrations ranging from 15.625 to 1000 µg mL^−1^. Each well was inoculated with the corresponding bacterial suspension prepared from a 0.5 McFarland standard. Growth-control wells containing the bacterial inoculum without treatment and sterility-control wells containing uninoculated broth were included. Following incubation at 37 °C for 24 h, the MIC was defined as the lowest concentration showing no visible bacterial growth. To determine the minimum bactericidal concentration (MBC), aliquots from wells showing no visible growth were inoculated onto Mueller–Hinton agar plates and incubated at 37 °C for an additional 24 h. The MBC was defined as the lowest tested concentration that produced no visible colony growth.

#### 2.6.2 Quantification of biofilm inhibition

The antibiofilm activities of the monometallic AgNPs, CuONPs, and ZnONPs and bimetallic Ag–CuONPs and Ag–ZnONPs were evaluated against *Staphylococcus aureus* ATCC 35556 and *Pseudomonas aeruginosa* ATCC 9027 using the microtiter-plate biofilm assay, as previously described by.^20^*Plantago ovata* extract and the corresponding metal precursors, AgNO_3_, Cu(NO_3_)_2_, and Zn(NO_3_)_2_, were evaluated in parallel as comparator groups to distinguish the antibiofilm effects of the synthesized nanomaterials from those of the free extract and metal salts. Untreated bacterial cultures served as the biofilm-growth control.^22^ Overnight bacterial cultures were diluted (1%) glucose-supplemented TS broth media. Aliquots of this suspension were then moved to a MTP 96-well containing a gradient of nanoparticle concentrations (15.625–1000 µg mL^−1^). Subsequently 48 h of constant condition at 37 °C, all cells were eliminated *via* the solution of PBS (pH 7.4). Then, the remaining adherent biofilms were fixed with 95% methanol, stained with 0.3% crystal violet, and destained with 30% acetic acid. The Abs of the solubilized crystal violet was measured at 540 nm, providing a spectrophotometric measure of the residual adherent biofilm biomass.

#### 2.6.3 Membrane integrity assessment *via* protein leakage assay

The effect of *Plantago ovata*-mediated Ag–CuO and Ag–ZnO nanoparticles on bacterial membrane integrity was evaluated by measuring extracellular protein leakage in *S. aureus* ATCC 35556 and *P. aeruginosa* ATCC 9027. Bacterial suspensions were adjusted to 0.5 McFarland standard (∼1 × 10^8^ CFU mL^−1^) and exposed to different concentrations of the synthesized nanoparticles in nutrient broth, while untreated cultures served as controls. Following incubation at 37 °C for 5 h, the cultures were centrifuged at 5500 rpm for 15 min to obtain cell-free supernatants. Protein leakage was determined by mixing 100 µL of the supernatant with 1 mL of Bradford reagent, followed by incubation in the dark for 10 min. The absorbance was measured at 595 nm using a UV-vis spectrophotometer. Increased absorbance values were interpreted as an indication of enhanced membrane disruption and leakage of intracellular proteins caused by nanoparticle treatment.

#### 2.6.4 Gene expression analysis of virulence-related genes

The anti-virulence potential of *Plantago ovata*-derived Ag–CuO and Ag–ZnO nanoparticles was evaluated by analyzing the expression of selected virulence-associated genes. Bacterial cultures were treated with a sub-inhibitory concentration (½ MIC) to assess the effect on virulence factors without affecting bacterial growth. Total RNA was extracted from treated and untreated cultures using TRIzol reagent, followed by complementary DNA (cDNA) synthesis using a reverse transcription kit according to the manufacturer's instructions. Gene expression analysis targeted *FnbA* and *cna* in *S. aureus* and *algD* and *lasB* in *P. aeruginosa*, with recA used as a housekeeping gene for normalization. Primer sequences are listed in Table S2. PCR amplification products were initially confirmed by agarose gel electrophoresis, and quantitative analysis was performed using real-time PCR (qRT-PCR) with SYBR Green chemistry. Relative gene expression levels were calculated by comparing treated samples to untreated controls, demonstrating the inhibitory effects of the synthesized nanoparticles on bacterial virulence and biofilm-associated genes.

### 2.7 Assessment of cell metabolic activity and *in vitro* anticancer effects

#### 2.7.1 Cell culture conditions

Caco-2 colorectal adenocarcinoma cells (ATCC HTB-37), MCF-7 breast adenocarcinoma cells (ATCC HTB-22), and Vero kidney epithelial cells (ATCC CCL-81) were obtained from the American Type Culture Collection. For the MTT assay, the three cell lines were cultured and treated in serum-supplemented RPMI-1640 medium under standard culture conditions at 37 °C in a humidified atmosphere containing 5% CO_2_. For the subsequent flow-cytometric and gene-expression experiments, Caco-2 cells were cultured in DMEM supplemented with 10% fetal bovine serum and 1% penicillin–streptomycin–amphotericin B solution, as specified in the corresponding experimental sections.

#### 2.7.2 MTT cell metabolic-activity assay

The effects of the monometallic AgNPs, CuONPs, and ZnONPs and bimetallic Ag–CuONPs and Ag–ZnONPs on cellular metabolic activity were evaluated using the MTT assay. Two human cancer cell lines, Caco-2 colorectal adenocarcinoma cells (ATCC HTB-37) and MCF-7 breast adenocarcinoma cells (ATCC HTB-22), were examined. Vero kidney epithelial cells (ATCC CCL-81) were used as a non-malignant comparator to evaluate the relative selectivity of the tested materials. Cells were seeded into 96-well plates at a density of 1 × 10^5^ cells per mL in serum-supplemented RPMI-1640 medium and incubated for 24 h to allow cell attachment. The culture medium was then removed, and the cell monolayers were gently washed with phosphate-buffered saline. The cells were subsequently treated for 48 h with AgNPs, CuONPs, ZnONPs, Ag–CuONPs, or Ag–ZnONPs over a concentration range of 15.625–1000 µg mL^−1^ prepared in the same serum-supplemented RPMI-1640 medium used for the MTT assay. *Plantago ovata* extract and the corresponding metal precursors, AgNO_3_, Cu(NO_3_)_2_, and Zn(NO_3_)_2_, were evaluated over the same concentration range as comparator groups, while doxorubicin was included as a reference anticancer agent. Untreated cells containing culture medium alone served as the negative control.

Following the 48 h treatment period, the treatment medium was removed, and the cells were incubated with MTT solution at a concentration of 5 mg mL^−1^ for 4 h to permit the formation of formazan crystals by metabolically active cells. The MTT-containing medium was then aspirated, and the resulting formazan crystals were dissolved in dimethyl sulfoxide. Absorbance was measured at 560 nm using a microplate reader. Cell metabolic activity was expressed as a percentage relative to the untreated control according to the following equation:Cell metabolic activity (%) = (absorbance of treated cells/absorbance of untreated control cells) × 100.

#### 2.7.3 Apoptosis and cell cycle analysis by flow cytometry

Apoptosis in Caco-2 cells was assessed using Annexin V/PI staining following treatment with the respective IC_50_ concentrations of Ag–CuONPs and Ag–ZnONPs. Cells were seeded into six-well plates at a density of 1 × 10^6^ cells per well in DMEM supplemented with 10% fetal bovine serum and 1% penicillin–streptomycin–amphotericin B solution and incubated at 37 °C in a humidified atmosphere containing 5% CO_2_ for 24 h. The culture medium was then replaced with fresh medium containing the respective nanomaterial at its IC_50_ concentration, while vehicle-treated cells served as the negative control. After 48 h, the cells were harvested using 0.25% trypsin–EDTA, washed twice with PBS, and adjusted to 1 × 10^6^ cells per mL. For apoptosis analysis, the cells were stained using the Alexa Fluor® 488 Annexin V/Dead Cell Apoptosis Kit (Invitrogen, Thermo Fisher Scientific; Cat. No. V13241). Briefly, 1 × 10^6^ cells were resuspended in 100 µL of 1× Annexin-binding buffer and incubated at room temperature for 5 min. Alexa Fluor® 488 Annexin V (5 µL) and PI working solution (1 µL, 100 µg mL^−1^) were then added, and the samples were incubated for 15 min at room temperature in the dark. Subsequently, 400 µL of 1× Annexin-binding buffer was added, and the samples were analyzed using a Beckman Coulter Navios EX flow cytometer. Fluorescence emissions were measured at approximately 530 and 575 nm following excitation at 488 nm. Cells were classified according to the quadrant definitions applied by the analysis software as viable, early apoptotic, late apoptotic, or necrotic populations. For cell-cycle analysis, separate treated and control cell samples were harvested, washed, and resuspended in complete DMEM at a concentration of 1 × 10^6^ cells per mL. One millilitre of each cell suspension was stained with 1 µL of Vybrant® DyeCycle™ Violet stain (Invitrogen, Thermo Fisher Scientific; Cat. No. V35003), producing a final dye concentration of 5 µM. Samples were incubated at 37 °C for 30 min in the dark, maintained at 37 °C until acquisition, and analyzed without subsequent washing or fixation using approximately 405 nm excitation and 440 nm emission. DNA-content histograms were analyzed using Navios EX software to determine the distributions of cells across the reported cell-cycle phases.

#### 2.7.4 Gene expression analysis by quantitative real-time PCR (RT-qPCR)

The expression levels of apoptosis-related genes (caspase-3, *BAX*, and *Bcl-2*) in Caco-2 (colorectal adenocarcinoma) cells treated with *Plantago ovata*-mediated Ag–CuO and Ag–ZnO nanoparticles at the IC_50_ concentration were evaluated using quantitative real-time PCR (RT-qPCR). Total RNA was extracted from both treated and untreated (control) cells using TRIzol reagent, and RNA purity and concentration were determined using a NanoDrop spectrophotometer. Complementary DNA (cDNA) was synthesized from 1 µg of total RNA using a reverse transcription kit according to the manufacturer's instructions. Quantitative PCR was carried out using a StepOnePlus™ Real-Time PCR System with SYBR Green Master Mix. Gene-specific primers (listed in Table S3) were employed, with β-actin used as the internal housekeeping gene for normalization. The amplification protocol consisted of an initial denaturation step followed by repeated cycles of denaturation, annealing, and extension. Relative gene expression levels were calculated using the 2^(−ΔΔCt)^ method. All experiments were performed in triplicate, and the results were expressed as mean ± standard deviation (SD).

### 2.8 Assessment of antioxidant activity

The radical-scavenging activities of the monometallic AgNPs, CuONPs, and ZnONPs and bimetallic Ag–CuONPs and Ag–ZnONPs were evaluated using complementary DPPH and ABTS assays. *Plantago ovata* extract and the corresponding metal precursors, AgNO_3_, Cu(NO_3_)_2_, and Zn(NO_3_)_2_, were evaluated in parallel as comparator groups. Ascorbic acid was used as the reference antioxidant.

For the DPPH assay, the tested samples were prepared over a concentration range of 15.625–1000 µg mL^−1^ and mixed with freshly prepared 1 mM DPPH solution in methanol. The reaction mixtures were incubated for 30 min in darkness, after which absorbance was measured at 517 nm. DPPH radical-scavenging activity was expressed as the percentage reduction in absorbance relative to the DPPH control^23^ and was calculated according to the following equation: DPPH radical-scavenging activity (%) = [(*A*_0_ − *A*_s_)/*A*_0_] × 100,where *A*_0_ is the absorbance of the DPPH control and *A*_s_ is the absorbance measured in the presence of the tested sample.

The ABTS radical-cation scavenging assay was performed according to the established protocol described in ref. 24. The ABTS radical cation was generated by mixing 7 mM ABTS solution with 2.4 mM potassium persulfate and incubating the mixture for 12–16 h in darkness. Before use, the resulting ABTS radical solution was diluted to an absorbance of 0.70 ± 0.02 at 734 nm. The diluted solution was then mixed with the tested samples at concentrations ranging from 15.625 to 1000 µg mL^−1^ and incubated at 30 °C for 10 min. Absorbance was subsequently recorded at 734 nm, and ABTS radical-cation scavenging activity was calculated using the following equation: ABTS radical-cation scavenging activity (%) = [(*A*_0_ − *A*_s_)/*A*_0_] × 100,where *A*_0_ is the absorbance of the ABTS radical control and *A*_s_ is the absorbance measured in the presence of the tested sample.

### 2.9 Statistical analysis

All experiments were performed in three independent replicates, and quantitative data are presented as mean ± standard deviation. Depending on the experimental design, comparisons involving a single factor were analyzed using one-way analysis of variance, whereas experiments involving treatment type and concentration were analyzed using two-way analysis of variance. Tukey's multiple-comparisons test was applied where appropriate. Statistical analyses were performed using Origin software, and differences were considered statistically significant at *p* < 0.05. GraphPad Prism version 8.0 was used to generate graphical representations of the biological data. IC_50_ values were calculated separately for each independent replicate by linear interpolation between the two consecutive tested concentrations that bracketed 50% inhibition, and the resulting values are reported as mean ± standard deviation. Microscopy-based particle-size measurements and image quantification were performed using ImageJ software.

## 3 Results and discussion

### 3.1 Phytochemical profiling of *Plantago ovata* seeds by HPLC

**Fig. 2:**
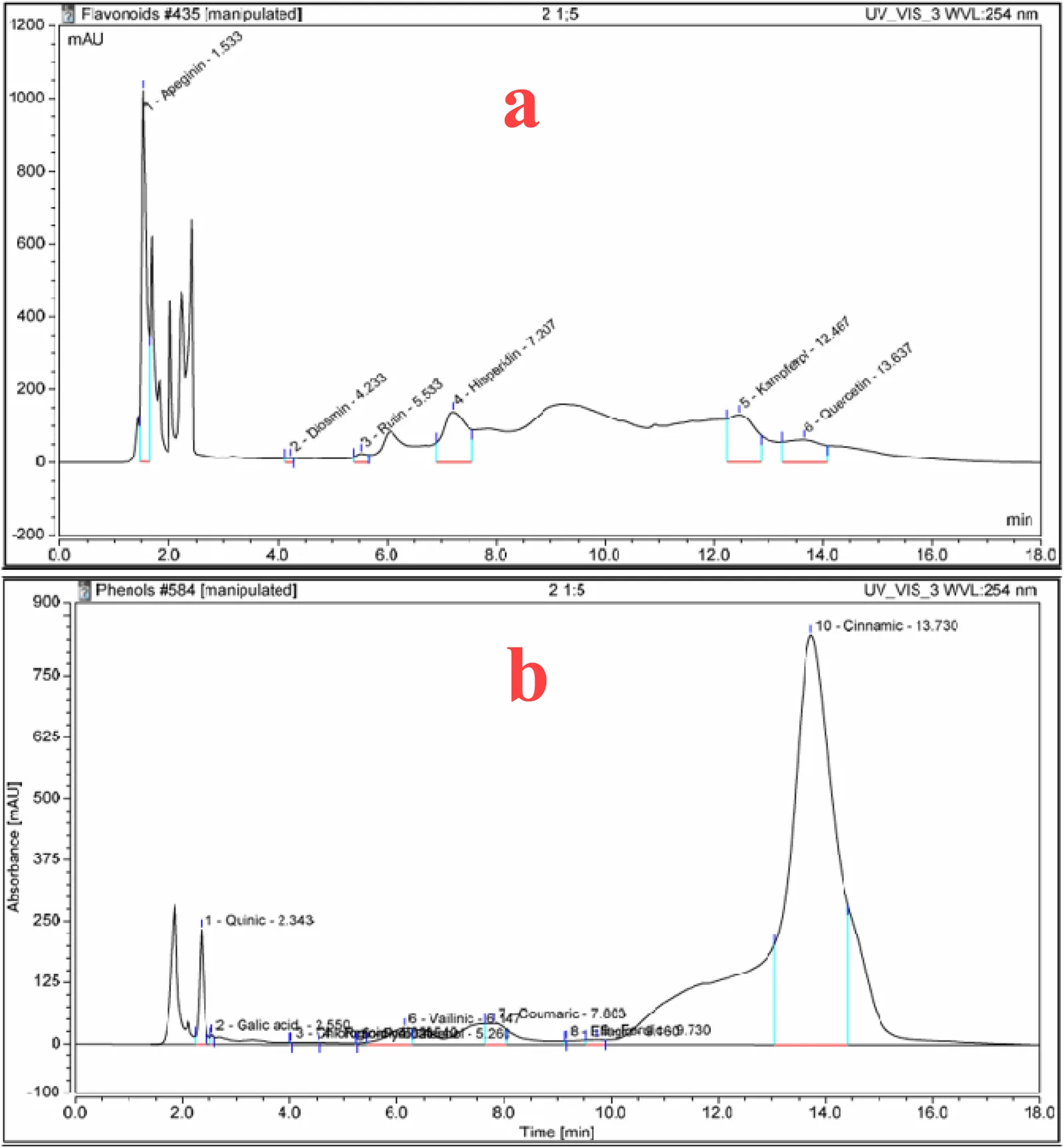
HPLC chromatograms of (a) flavonoids and (b) phenolic compounds in *Plantago ovata* seeds.

HPLC analysis of a representative *Plantago ovata* seed extract revealed a diverse profile of phenolic acids and flavonoids (Table 1 and Fig. 2). Apigenin was the predominant flavonoid (5086.82 µg g^−1^), followed by hesperidin (154.43 µg g^−1^), quercetin (149.21 µg g^−1^), and kaempferol (60.13 µg g^−1^), while rutin and diosmin were detected at lower concentrations. Among the phenolic compounds, cinnamic acid was the most abundant (6385.35 µg g^−1^), followed by quinic acid (593.45 µg g^−1^), vanillic acid (117.10 µg g^−1^), and coumaric acid (74.28 µg g^−1^). Gallic, chlorogenic, ellagic, and other phenolic acids were detected at lower concentrations. Phenolic acids and flavonoids contain hydroxyl, carbonyl, and carboxyl functional groups that may participate in metal-ion coordination and influence nucleation and particle growth during plant-mediated nanomaterial synthesis.^25–27^ In particular, the abundance of apigenin and cinnamic acid supports the suitability of *P. ovata* extract as a phytochemical-rich synthesis medium. Previous studies have reported that the composition and concentration of plant-derived phenolics can influence nanoparticle formation, size distribution, and colloidal behavior.^28^ However, the present HPLC analysis identifies the phytochemical constituents of the representative extract but does not independently establish the contribution of individual compounds to reduction or stabilization, nor does it confirm the persistence of a phytochemical coating after calcination. Therefore, these constituents are considered potential contributors to precursor coordination, nucleation, and stabilization during synthesis rather than confirmed reducing, capping, or functionalizing agents in the final nanomaterials.

### 3.2 Batch-to-batch reproducibility of *Plantago ovata* extract

Three independent *Plantago ovata* aqueous extract batches (E1–E3) were prepared under identical conditions to assess the reproducibility of the extraction procedure. As shown in Table S4, the pH values ranged from 6.20 to 6.63, with a mean of 6.48 ± 0.24 and a CV of 3.71%. The dry-solids concentrations ranged from 7.56 to 8.78 mg mL^−1^, giving a mean of 8.33 ± 0.67 mg mL^−1^ and a CV of 8.04%. Similarly, the extraction yields ranged from 15.12 to 17.56%, with a mean of 16.66 ± 1.34% and a CV of 8.04%. These relatively low CV values indicate limited batch-to-batch variability and support the reproducibility of the applied extraction procedure. The close pH values suggest that the chemical environments of the independently prepared extracts were broadly comparable. This consistency is relevant to plant-mediated nanomaterial synthesis because pH can influence phytochemical ionization, metal-ion reduction and coordination, nucleation, particle growth, and colloidal stability.^29^ The comparable dry-solids concentrations and extraction yields further indicate that similar amounts of water-soluble seed constituents were recovered under the controlled conditions. Previous research on *P. ovata* seeds has shown that extraction yield and phytochemical recovery can be affected by operating parameters such as temperature, extraction method, solvent characteristics, and particle size.^30^ The limited variation observed among E1–E3 may therefore reflect the natural heterogeneity of the plant material and minor experimental differences during mixing, filtration, or recovery of soluble constituents. Standardization of the plant material and extraction protocol is particularly important in plant-assisted nanomaterial synthesis because variation in extract preparation can influence the consistency and physicochemical characteristics of the resulting materials.^31^

### 3.3 Physicochemical characterization of the biosynthesized monometallic and bimetallic nanomaterials

#### 3.3.1 Optical, functional-group, and crystalline-phase analyses

**Fig. 3:**
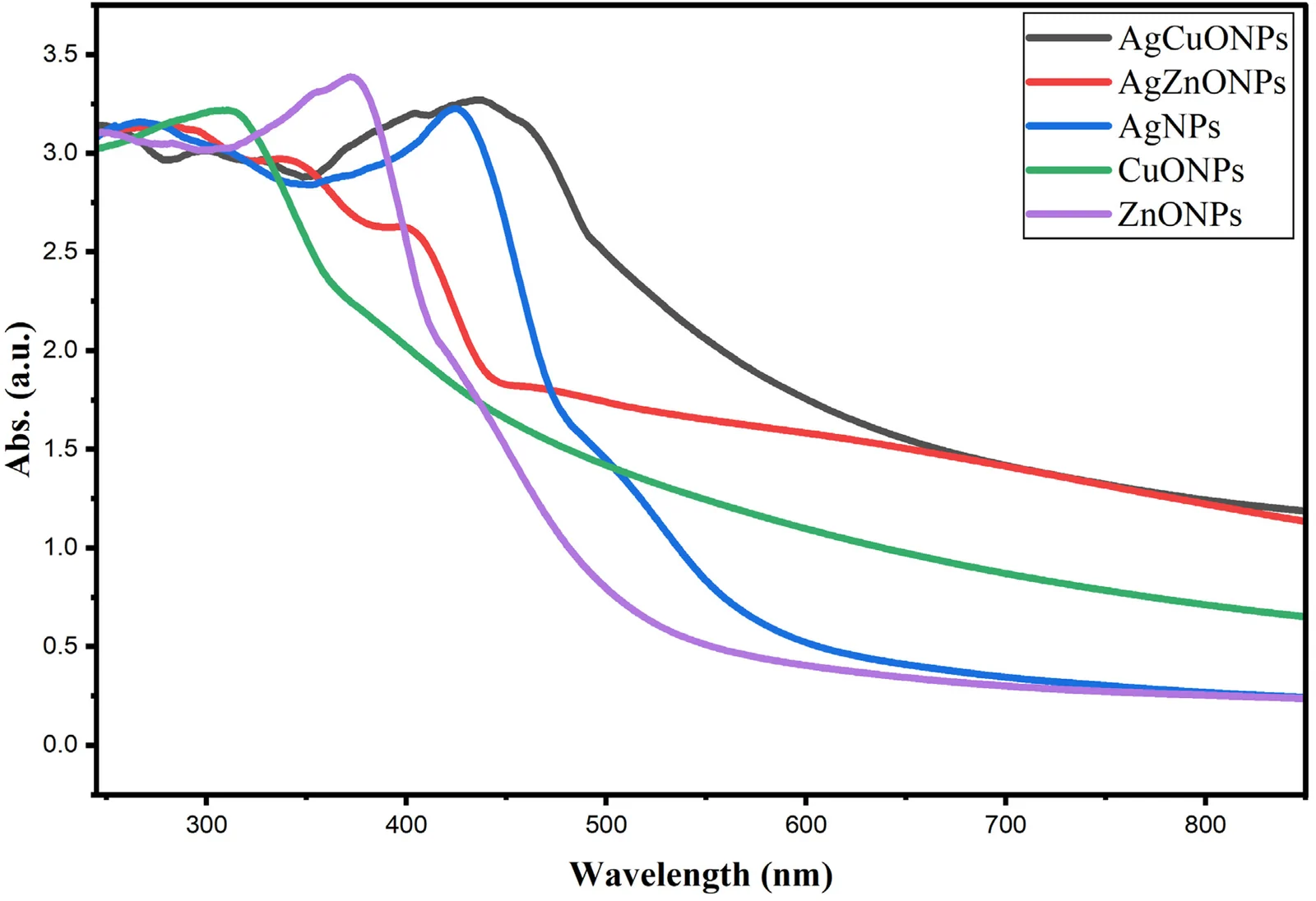
UV-vis absorption spectra of the green-synthesized Ag–CuO and Ag–ZnO nanomaterials and their corresponding monometallic AgNPs, CuONPs, and ZnONPs.

As shown in Fig. 3, the target Ag–CuONPs and Ag–ZnONPs exhibited optical responses that differed from those of the corresponding monometallic AgNPs, CuONPs, and ZnONPs used as comparative materials. Ag–CuONPs displayed absorption features at approximately 278 and 437 nm. The visible band at 437 nm was close to, but broader than, the AgNPs plasmonic band observed at 424 nm and was therefore attributed primarily to the optical response of the Ag component within the Ag–CuO mixed-phase material. Its broadening and displacement may reflect differences in particle size, aggregation, the local dielectric environment surrounding Ag, and possible interfacial contact with CuO. Ag–ZnONPs exhibited absorption features at approximately 337 and 396 nm. The band at 337 nm was assigned to the ZnO-related optical response, consistent with a recently reported green-synthesized Ag–ZnO system exhibiting a characteristic ZnO band at the same wavelength.^32^ The additional feature at 396 nm was tentatively associated with the Ag-containing component and modification of the optical environment following combined synthesis. Comparable changes in absorption-edge position and broadening of the UV-visible response have been reported for Ag-containing ZnO nanocomposites relative to pristine ZnO.^33^ The corresponding monometallic materials provided reference optical profiles for comparison. AgNPs exhibited a characteristic localized surface plasmon resonance band at 424 nm, consistent with recent green-synthesis studies reporting AgNPs absorption maxima at approximately 420–421 nm.^34,35^ CuONPs and ZnONPs displayed absorption bands at 316 and 373 nm, respectively, within the ultraviolet region commonly reported for nanoscale metal oxides. Plant-derived ZnONPs have similarly been reported to exhibit absorption maxima within approximately 362–375 nm.^34^ Variations in band positions may arise from differences in crystallite size, morphology, structural defects, aggregation, synthesis conditions, surface-associated species, and the dielectric properties of the surrounding medium. Overall, comparison with the monometallic materials demonstrated that the Ag–CuO NPs and Ag–ZnO NPs formulations possessed modified optical profiles following combined synthesis. Nevertheless, UV-visible spectroscopy alone cannot establish their phase composition or interfacial arrangement. Accordingly, these optical findings were considered complementary and were interpreted together with the XRD, HR-TEM lattice-fringe, EDX, and elemental-mapping results.

**Fig. 4:**
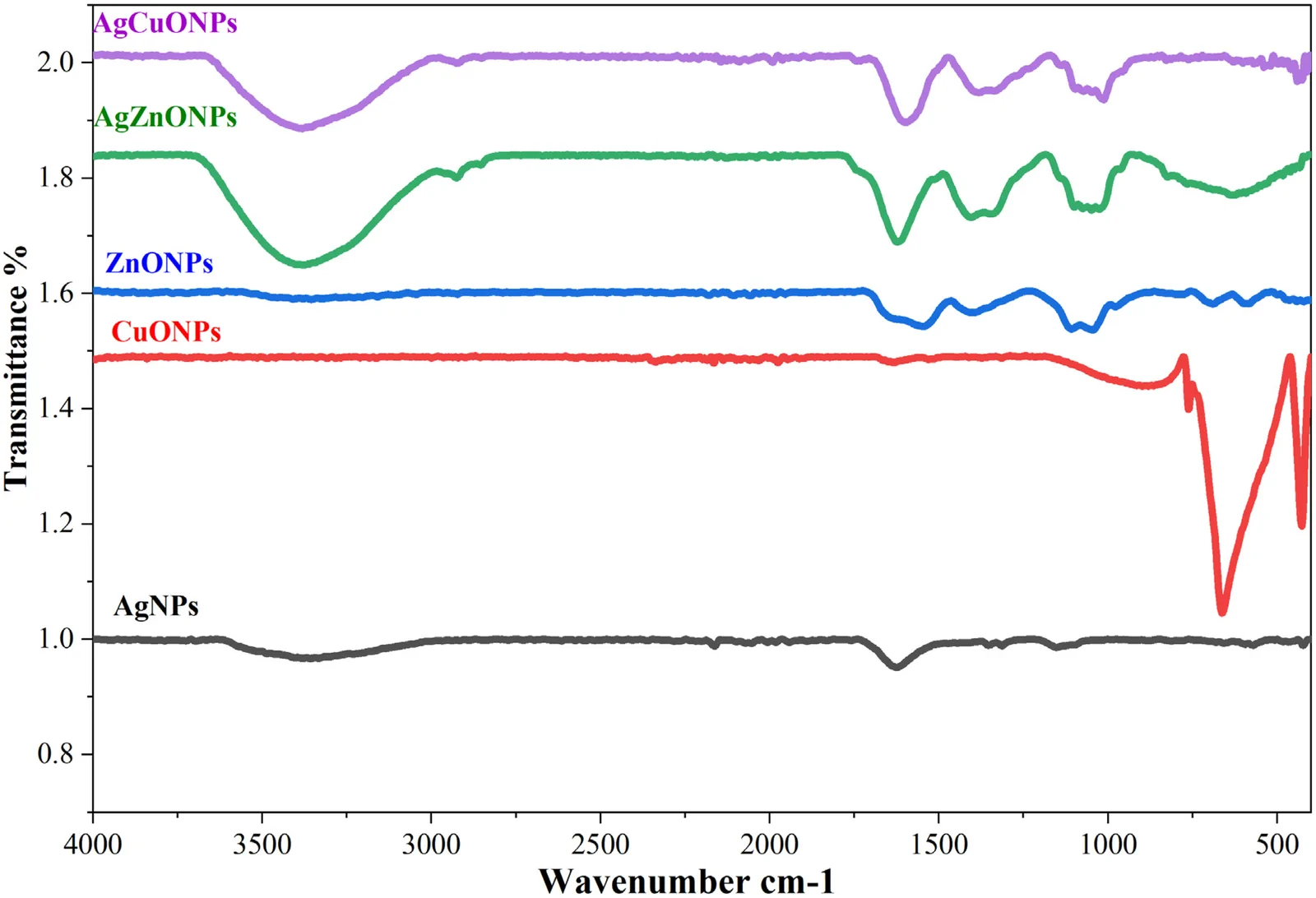
FTIR spectra of the green-synthesized Ag–CuO and Ag–ZnO nanomaterials and their corresponding monometallic AgNPs, CuONPs, and ZnONPs, recorded over the wavenumber range of 4000–400 cm^−1^. The spectra were vertically offset for clarity.

The FTIR spectra of the target Ag–CuONPs and Ag–ZnONPs, together with those of the corresponding monometallic AgNPs, CuONPs, and ZnONPs used as comparative materials, are presented in Fig. 4. Ag–CuONPs exhibited a broad O–H-associated band at approximately 3356 cm^−1^, weak C–H stretching features within 2938–2851 cm^−1^, and prominent bands at 1598, 1383–1337, 1272, and 1137–1013 cm^−1^. The lower-wavenumber features within approximately 680–426 cm^−1^ were primarily associated with Cu–O lattice vibrations. Comparable functional-group and metal–oxygen vibrational regions have been reported for plant-mediated Ag–CuO materials.^15^ For comparison, CuONPs displayed broad O–H-associated absorption at approximately 3471–3375 cm^−1^ and additional bands at 1592, 1366, and 1025 cm^−1^. The prominent band at 612 cm^−1^ and lower-wavenumber features near 471 and 438 cm^−1^ were assigned to Cu–O lattice vibrations. AgNPs exhibited a broad O–H-associated band cantered at approximately 3358 cm^−1^ and a prominent band at 1624 cm^−1^, which may be associated with carbonyl-containing surface species and/or the bending vibration of adsorbed water. Additional bands at approximately 1445, 1353, 1313, and 1153 cm^−1^ indicated the presence of oxygen-containing surface groups. Similar O–H-, carbonyl-, C–O-, and Cu–O-associated regions have been identified in green-synthesized metal and metal oxide nanomaterials.^36^ Ag–ZnONPs exhibited broad O–H-associated absorption at approximately 3380–3346 cm^−1^, weak C–H stretching bands near 2925 and 2855 cm^−1^, and additional features at 1623, 1515, 1405, 1347, and 1100–1026 cm^−1^. Bands below 700 cm^−1^, including those near 689, 632, 610, 592, 561, 530, 497, 462, and 434 cm^−1^, were generally attributed to metal–oxygen lattice vibrations, principally those associated with the Zn–O phase. Related vibrational features have been reported for green-synthesized Ag-modified ZnO nanomaterials.^37^ The corresponding ZnONPs displayed a broad O–H-associated region at approximately 3379–3323 cm^−1^, together with bands at 1545, 1398, 1108, and 1045 cm^−1^ that may originate from carbonyl-, hydroxyl-, and C–O-containing surface species. Bands at approximately 584, 498, and 440 cm^−1^ were consistent with Zn–O lattice vibrations previously reported for green-synthesized ZnO and Ag-modified ZnO materials.^38^ Comparison with the monometallic materials revealed differences in the positions and profiles of several bands in the Ag–CuONPs and Ag–ZnONPs spectra. These differences may reflect changes in surface coordination and the local bonding environment following combined synthesis, as observed in comparative studies of plant-mediated Ag, CuO, and Ag–CuO materials.^39^ The O–H-, carbonyl-, and C–O-associated bands suggest the presence of surface hydroxyl groups, adsorbed water, and residual oxygen-containing surface species. However, because the samples were calcined at 400 °C, these bands should not be regarded as definitive evidence of an intact phytochemical capping layer. FTIR analysis also cannot independently establish the phase composition or interfacial arrangement of the Ag–CuO and Ag–ZnO materials. Accordingly, the FTIR findings were interpreted together with the XRD, HR-TEM, EDX, and elemental-mapping results.

**Fig. 5:**
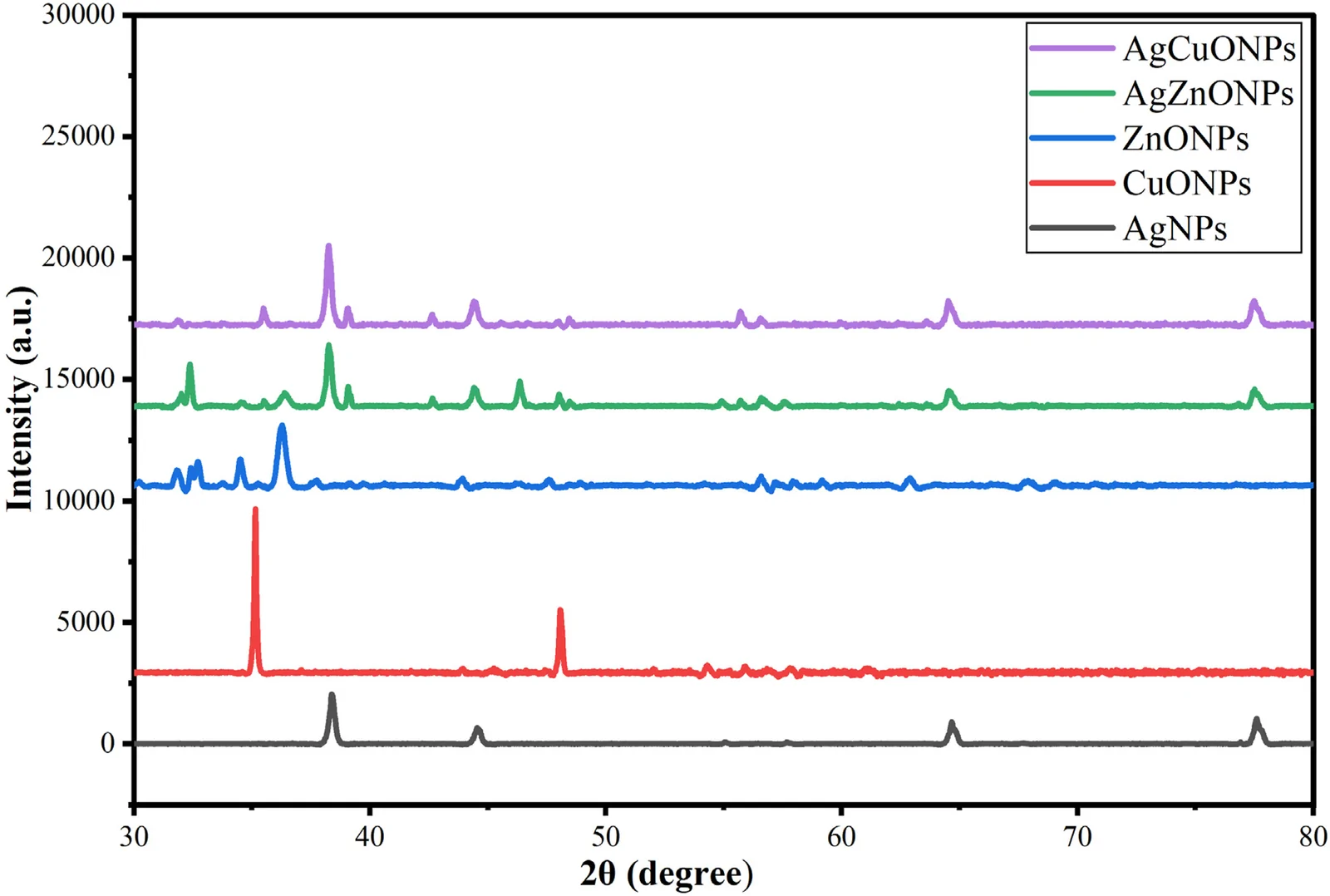
Stacked X-ray diffraction patterns of the biosynthesized Ag–CuO and Ag–ZnO nanomaterials compared with the corresponding monometallic AgNPs, CuONPs, and ZnONPs. The patterns were vertically offset for clarity. Ag–CuO and Ag–ZnO exhibited diffraction reflections attributable to the coexistence of Ag with the respective CuO and ZnO crystalline phases.

The XRD patterns of the Ag–CuO and Ag–ZnO nanomaterials and their corresponding monometallic comparators are presented in Fig. 5. A single reference card was consistently applied to each crystalline phase in both the monometallic and mixed-phase samples: PDF No. 01-071-3762 for face-centred cubic Ag, PDF No. 01-089-2531 for monoclinic CuO tenorite, and PDF No. 01-070-2551 for hexagonal wurtzite ZnO zincite. AgNPs exhibited characteristic reflections at 2*θ* = 38.42°, 44.60°, 64.60°, and 77.60°, indexed to the (111), (200), (220), and (311) planes, respectively, of face-centred cubic metallic Ag. CuONPs displayed reflections at 35.50°, 48.50°, 58.10°, and 61.50°, assigned to the (−111), (−202), (202), and (−113) planes, respectively, of monoclinic CuO. These assignments are consistent with previously reported Ag and CuO crystalline structures.^15,40^ ZnONPs showed diffraction peaks at 31.80°, 34.50°, 36.20°, 47.80°, 56.60°, and 62.69°, corresponding to the (100), (002), (101), (102), (110), and (103) planes, respectively, of hexagonal wurtzite ZnO, in agreement with previously reported ZnO diffraction patterns.^41^ The Ag–CuO pattern exhibited reflections at 35.50°, 38.25°, 44.40°, 64.50°, and 77.50°. The reflection at 35.50° was assigned to the (−111) plane of monoclinic CuO, whereas those at 38.25°, 44.40°, 64.50°, and 77.50° corresponded to the (111), (200), (220), and (311) planes, respectively, of face-centred cubic Ag. The reflection around 38.25° may contain overlapping contributions from the Ag (111) and CuO (111) planes. The relatively low intensity or absence of other distinct CuO reflections may be associated with peak overlap, reduced CuO crystallinity, small crystallite dimensions, or its relative abundance within the mixed-phase material. The Ag–ZnO pattern displayed reflections at 31.90°, 34.50°, 36.20°, 38.25°, 44.40°, 48.00°, 56.70°, 64.60°, and 77.50°. The reflections at 31.90°, 34.50°, 36.20°, 48.00°, and 56.70° were assigned to the (100), (002), (101), (102), and (110) planes, respectively, of hexagonal wurtzite ZnO. The reflections at 38.25°, 44.40°, 64.60°, and 77.50° corresponded to the (111), (200), (220), and (311) planes, respectively, of face-centred cubic Ag. Similar coexistence of Ag- and ZnO-related crystalline reflections has been reported for Ag-containing ZnO nanomaterials.^37,38^ Collectively, the simultaneous occurrence of Ag- and CuO-related reflections in Ag–CuO and Ag- and ZnO-related reflections in Ag–ZnO supports the coexistence of the corresponding crystalline phases within the synthesized mixed-phase nanomaterials. Nevertheless, XRD alone cannot establish their precise nanoscale distribution or interfacial arrangement. The phase assignments were therefore further evaluated using HR-TEM lattice-fringe analysis, EDX, and elemental mapping.

#### 3.3.2 Morphological and colloidal characterization

**Fig. 6:**
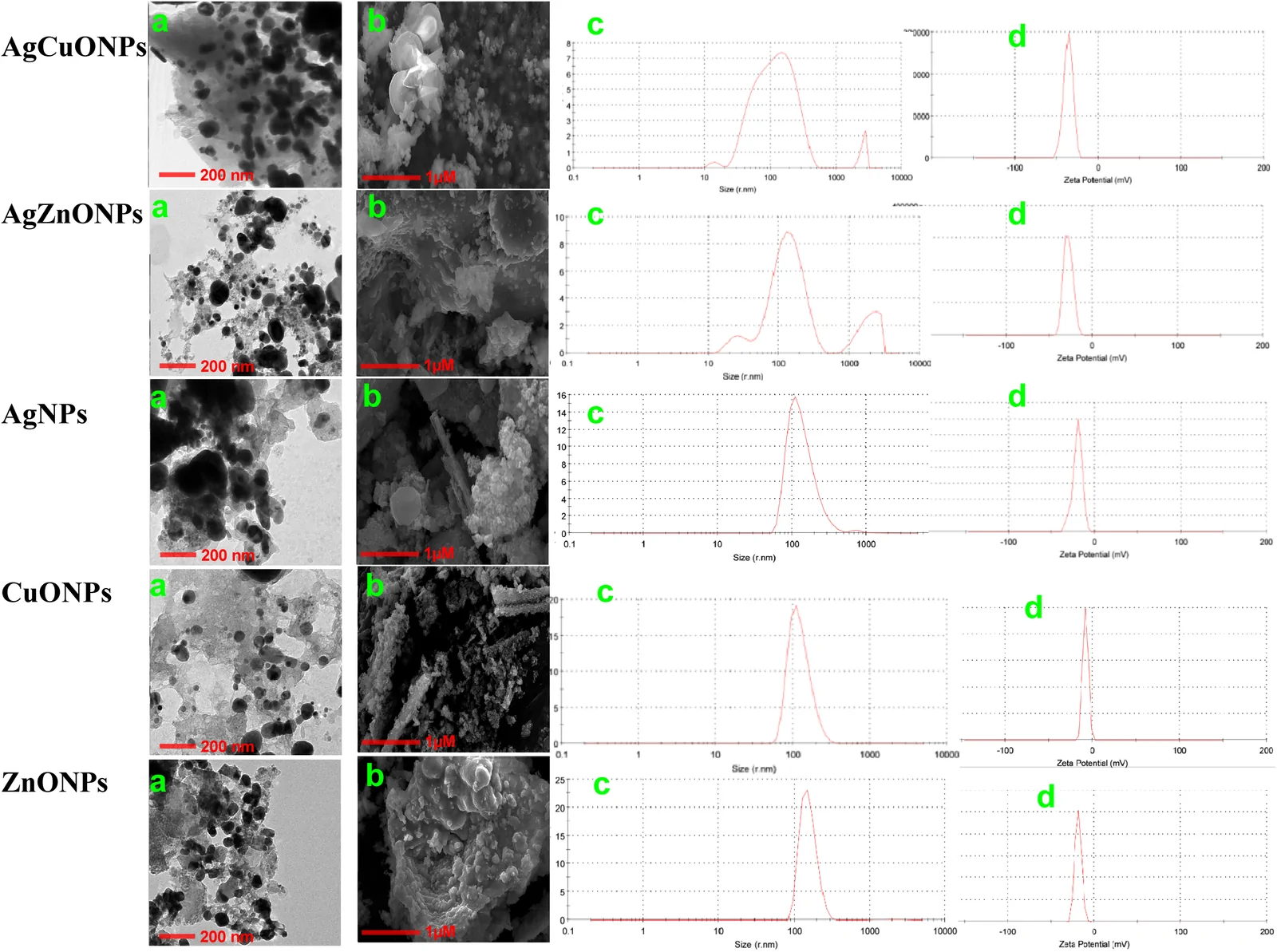
Comparative morphological and colloidal characterization of the biosynthesized Ag–CuONPs, Ag–ZnONPs, AgNPs, CuONPs, and ZnONPs. Panels show (a) TEM images, (b) SEM images, (c) hydrodynamic particle-size distributions measured by DLS, and (d) zeta-potential distributions. Scale bars are indicated within the corresponding TEM and SEM images.

Comparative TEM and SEM images and the corresponding DLS and zeta-potential distributions of the synthesized nanomaterials are presented in Fig. 6. TEM analysis revealed mean particle sizes of 72.02 ± 28.49, 83.30 ± 32.10, 41.60 ± 24.70, 32.87 ± 18.00, and 33.41 ± 9.60 nm for Ag–CuONPs, Ag–ZnONPs, AgNPs, CuONPs, and ZnONPs, respectively. The larger mean sizes and standard deviations of Ag–CuONPs and Ag–ZnONPs indicate broader primary-particle distributions than those of the monometallic CuONPs and ZnONPs. TEM images also revealed particle contact and localized aggregation, particularly within the mixed-phase samples. SEM examination showed clusters of different sizes and compactness across the five formulations. Such clustering may partly arise from interparticle interactions and capillary forces during drying and sample preparation and does not necessarily represent the dispersion state of the particles in the original aqueous suspensions. DLS analysis yielded Z-average hydrodynamic diameters of 103.8, 124.4, 668.9, 684.9, and 1301 nm for Ag–CuONPs, Ag–ZnONPs, AgNPs, CuONPs, and ZnONPs, respectively, with corresponding PDI values of 0.472, 0.574, 0.844, 1.000, and 0.574. The principal distribution modes occurred at 136.4, 146.2, 140.0, 125.5, and 154.5 nm, respectively. Additional populations were detected at 2584 and 14.15 nm for Ag–CuONPs and at 1926 and 26.78 nm for Ag–ZnONPs. Minor micron-sized populations were also observed at 912.1, 1591, and 1930 nm for AgNPs, CuONPs, and ZnONPs, respectively, demonstrating different degrees of aggregation in aqueous suspension. The hydrodynamic dimensions determined by DLS were larger than the corresponding TEM-derived sizes because the two techniques measure particles under different physical conditions. TEM measures dried particles under vacuum, whereas DLS determines the effective diffusion diameter of particles and aggregates dispersed in a liquid medium. Similar differences between TEM-derived particle sizes and DLS-derived hydrodynamic diameters have been reported for green-synthesized nanocomposites.^42^ The relatively closer correspondence between the TEM and DLS results for Ag–CuONPs and Ag–ZnONPs suggests better dispersion of the mixed-phase formulations than the monometallic samples. Nevertheless, their PDI values of 0.472 and 0.574 indicate moderately broad and broad distributions, respectively, rather than monodisperse suspensions. The substantially larger Z-average values obtained for AgNPs, CuONPs, and ZnONPs were associated with the presence of aggregates, to which light-scattering measurements are particularly sensitive. Hydrodynamic diameter can include the particle core, surface-associated layers, the surrounding solvation layer, and contributions from particle aggregation.^43^ In addition, the AgNPs, CuONPs, and ZnONPs measurements were flagged by the instrument for quality review and showed correlogram intercepts of 0.0748, 0.164, and 0.302, respectively. Their Z-average values should therefore be interpreted cautiously and should not be considered estimates of primary particle size. For these samples, the TEM-derived dimensions provide a more direct estimate of individual particle size, whereas DLS primarily demonstrates dispersion heterogeneity and aggregation under the applied measurement conditions. Zeta-potential analysis showed single negatively charged populations at −36.1, −28.1, −20.0, −8.15, and −18.9 mV for Ag–CuONPs, Ag–ZnONPs, AgNPs, CuONPs, and ZnONPs, respectively. Ag–CuONPs exhibited the highest surface-charge magnitude and therefore the strongest electrostatic contribution to colloidal stability among the tested formulations. Ag–ZnONPs showed an intermediate surface-charge magnitude, although their elevated PDI and secondary micron-sized population indicated that aggregation was not completely prevented. AgNPs and ZnONPs exhibited weaker electrostatic stabilization, whereas CuONPs showed the lowest surface-charge magnitude and the greatest tendency toward aggregation. However, zeta potential should be interpreted as an indicator of electrokinetic behaviour under the specified measurement conditions rather than as an absolute measure of stability, particularly for aggregated dispersions.^44^ Therefore, these findings should not be extrapolated directly to media with different pH, ionic strength, or protein content.

#### 3.3.3 HR-TEM lattice-fringe analysis

**Fig. 7:**
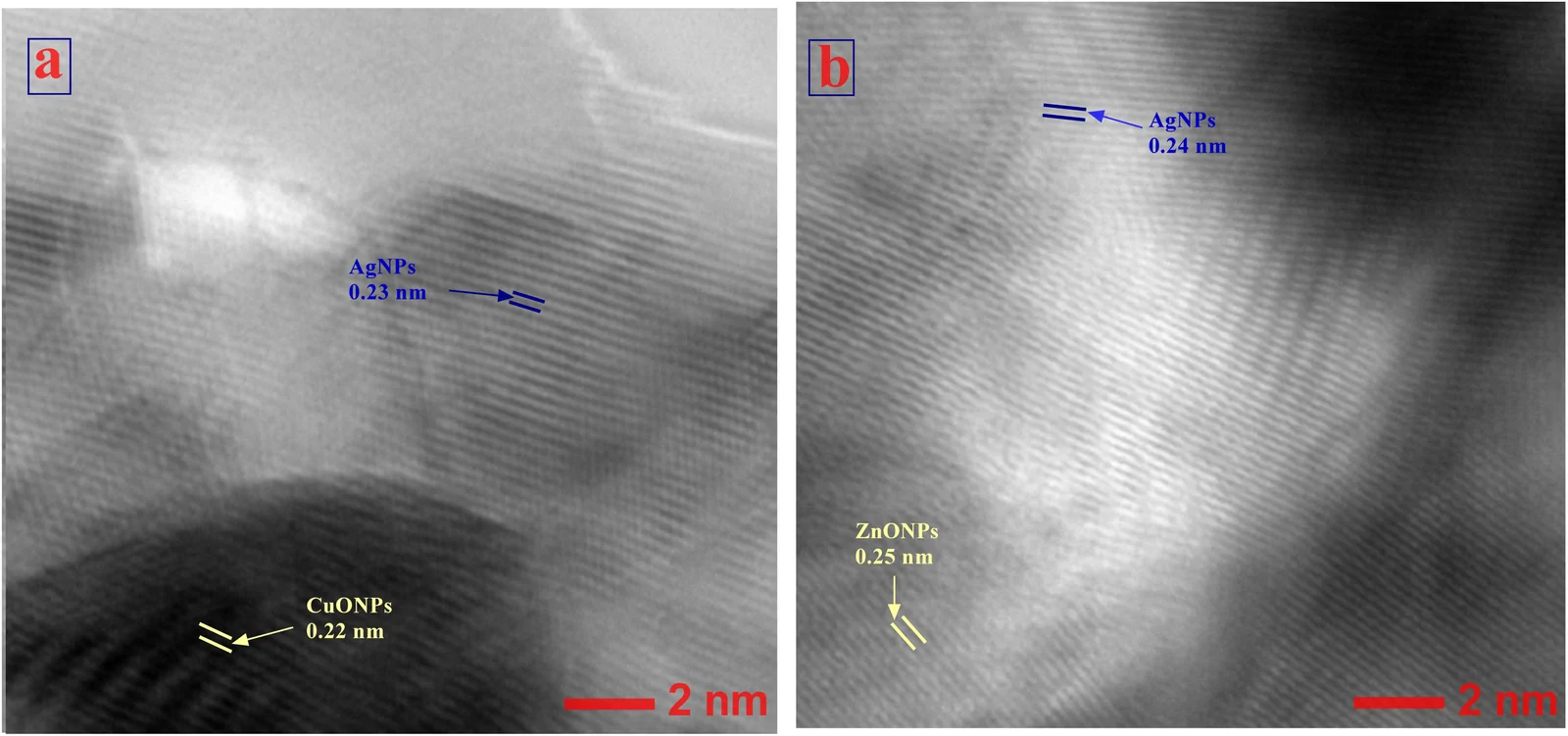
High-resolution transmission electron microscopy (HR-TEM) images of (a) Ag–CuONPs and (b) Ag–ZnONPs, showing well-resolved lattice fringes assigned to the corresponding Ag and metal-oxide crystalline phases.

HR-TEM examination revealed well-resolved lattice fringes within selected regions of both mixed-phase nanomaterials (Fig. 7), confirming their local crystalline character. In Ag–CuONPs, the measured lattice spacings of approximately 0.23 and 0.22 nm were consistent with highly crystalline domains. The spacing near 0.23 nm was consistent with the (111) plane of face-centred cubic Ag, whereas the 0.22 nm spacing was tentatively associated with a CuO-related domain, considering the independent identification of monoclinic CuO by XRD. Reported HR-TEM investigations have identified Ag (111) lattice spacings of approximately 0.23–0.24 nm, while the principal CuO lattice spacings commonly occur within approximately 0.23–0.25 nm.^45,46^ Because of the proximity and possible overlap of Ag- and CuO-related lattice spacings, the CuO assignment should be regarded as supportive rather than definitive. Ag–ZnONPs displayed lattice-fringe spacings of approximately 0.24 and 0.25 nm. The spacing near 0.24 nm was consistent with the (111) plane of face-centred cubic Ag, whereas the spacing of approximately 0.25 nm was assigned to the (101) plane of hexagonal wurtzite ZnO. Comparable lattice spacings have been reported for Ag-containing ZnO nanostructures.^47^ The presence of distinguishable lattice fringes supports the local coexistence of crystalline metal and metal-oxide domains within the examined regions. Together with the XRD phase assignments, EDX spectra, and elemental-mapping results, these HR-TEM observations provide complementary evidence supporting the formation of mixed-phase Ag–CuO and Ag–ZnO nanomaterials. However, HR-TEM examines highly localized regions and does not independently establish the bulk elemental composition, uniform phase distribution throughout the samples, oxidation states, or precise three-dimensional interfacial architecture.

#### 3.3.4 EDX and elemental mapping

**Fig. 8:**
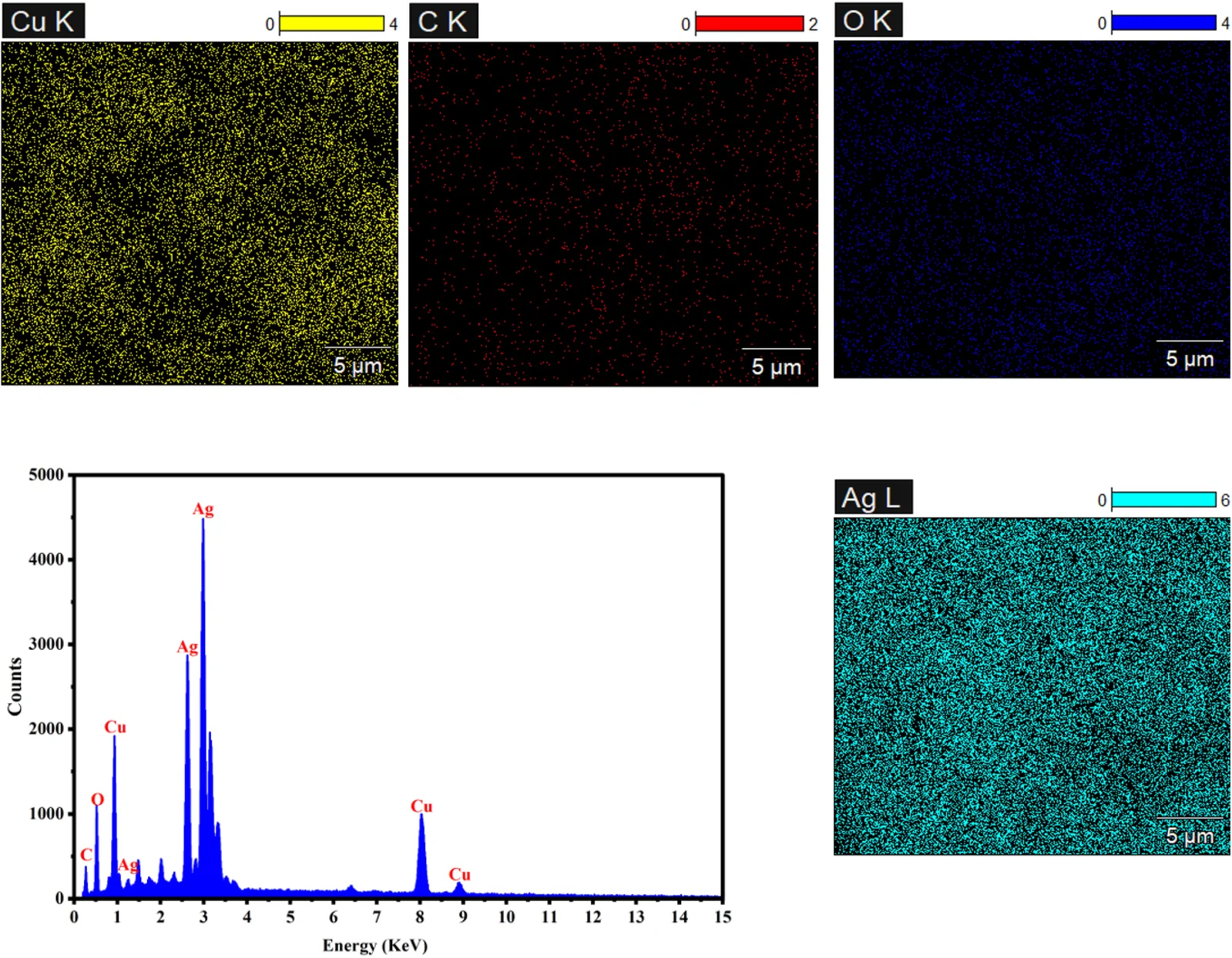
EDX spectrum and elemental mapping of the biosynthesized Ag–CuONPs.

**Fig. 9:**
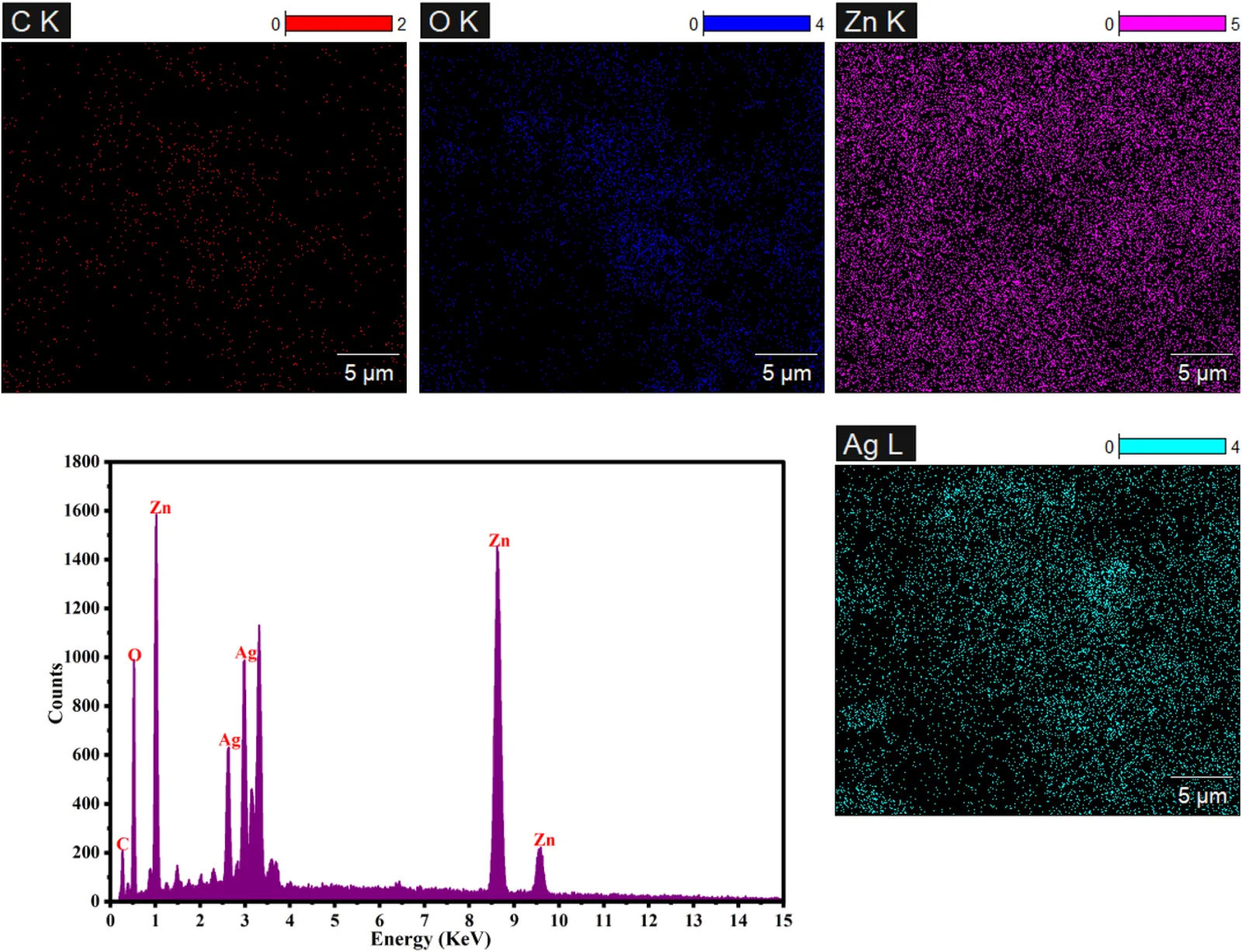
EDX spectrum and elemental mapping of the biosynthesized Ag–ZnONPs.

The elemental composition and micrometer-scale spatial distribution of the constituent elements in Ag–CuONPs and Ag–ZnONPs were investigated using EDX spectroscopy and elemental mapping, as presented in Fig. 8 and 9. The EDX spectrum of Ag–CuONPs showed distinct signals corresponding to Ag, Cu, and O, together with a weak carbon signal. Semi-quantitative analysis yielded weight percentages of 55.6% Ag, 19.4% Cu, 22.6% O, and 2.4% C, corresponding to atomic percentages of 21.2%, 12.5%, 58.1%, and 8.2%, respectively. The substantially higher weight percentage of Ag partly reflects its greater atomic mass and should not be interpreted directly as evidence of its molar predominance. Elemental mapping demonstrated that Ag, Cu, and O were broadly distributed throughout the analysed region, supporting the coexistence of Ag- and CuO-containing crystalline components in the Ag–CuO material. Comparable identification and spatial mapping of Ag, Cu, and O have recently been reported for Ag/CuO nanocomposites.^48^

For Ag–ZnONPs, the EDX spectrum contained the characteristic signals of Ag, Zn, and O, in addition to a minor carbon signal. The measured weight percentages were 20.9% Ag, 53.8% Zn, 21.4% O, and 3.9% C, whereas the corresponding atomic percentages were 7.2%, 30.7%, 49.9%, and 12.2%, respectively. Thus, Zn was the predominant metallic element within the analysed region. The elemental maps showed the broad distribution of Ag, Zn, and O across the selected field, supporting the coexistence of the Ag- and ZnO-containing components. Similar EDX signatures and elemental distributions have been reported for recently developed ZnO@Ag and green-synthesized Ag/ZnO nanocomposites.^49,50^ The oxygen signals detected in both formulations were consistent with the CuO and ZnO crystalline phases identified by XRD and HR-TEM. However, the presence of oxygen in an EDX spectrum does not independently establish the oxidation states of Cu or Zn. The weak carbon signals may originate from the carbon-based specimen support, adventitious carbon introduced during preparation, and/or residual carbonaceous surface species. Because the nanomaterials were calcined at 400 °C, the carbon signal alone cannot be considered conclusive evidence of an intact phytochemical coating. EDX elemental percentages should also be considered semi-quantitative because they may be influenced by the electron-beam interaction volume, specimen thickness, surface geometry, substrate contribution, and the region selected for analysis. Therefore, small differences in the reported elemental percentages should not be interpreted as precise bulk stoichiometric ratios. Nevertheless, the simultaneous detection of the expected elements and their distribution throughout the examined regions agreed with recent EDX analyses of plant-mediated Ag–ZnO materials.^51^

#### 3.3.5 Surface-area and pore analysis

Nitrogen adsorption–desorption isotherms were measured at 77 K for the biosynthesized Ag–CuONPs and Ag–ZnONPs (Fig. S1 and S2). The isotherms exhibited a characteristic Type IV profile with a distinct hysteresis loop in the intermediate relative pressure range (*P*/*P*_0_ = 0.4–0.9), indicating the presence of mesoporous structures and capillary condensation within interconnected pore networks according to the IUPAC classification.^52^ BET analysis revealed that the Ag–ZnO nanocomposites possessed a higher specific surface area (5.0685 m^2^ g^−1^) compared with Ag–CuO nanocomposites (3.0393 m^2^ g^−1^). The calculated BET C constants were 57.522 and 92.569 for Ag–ZnO and Ag–CuO, respectively, indicating strong interactions between nitrogen molecules and the surface adsorption sites of the metal oxide nanocomposites. Furthermore, Ag–ZnO exhibited a larger total pore volume (0.058691 cm^3^ g^−1^ at *P*/*P*_0_ = 0.99) compared with Ag–CuO (0.036466 cm^3^ g^−1^). The average pore diameter calculated using the BJH adsorption branch was 34.004 nm for Ag–ZnO and 49.239 nm for Ag–CuO, confirming the mesoporous nature of the synthesized nanocomposites. The observed differences in pore size and pore volume suggest variations in pore architecture and surface texture between the two nanocomposites. Such textural complexity is highly desirable for nanomaterials used in catalysis and adsorption, as it enhances molecular diffusion and accessibility to active sites.^53^ The BET results demonstrate differences in the surface area and pore characteristics of Ag–CuONPs and Ag–ZnONPs. The coexistence of the corresponding Ag and metal-oxide phases was established separately using XRD, HR-TEM, EDX, and elemental mapping. The significantly higher surface area and tailored porosity of Ag–ZnO, in particular, can be attributed to the bio-templating and capping action of the phytochemicals, which likely control nucleation and growth, preventing agglomeration and promoting a porous structure.^54^

### 3.4 Antibacterial and antibiofilm activities of Ag–CuONPs and Ag–ZnONPs

#### 3.4.1 Agar-diffusion and broth-dilution susceptibility

**Fig. 10:**
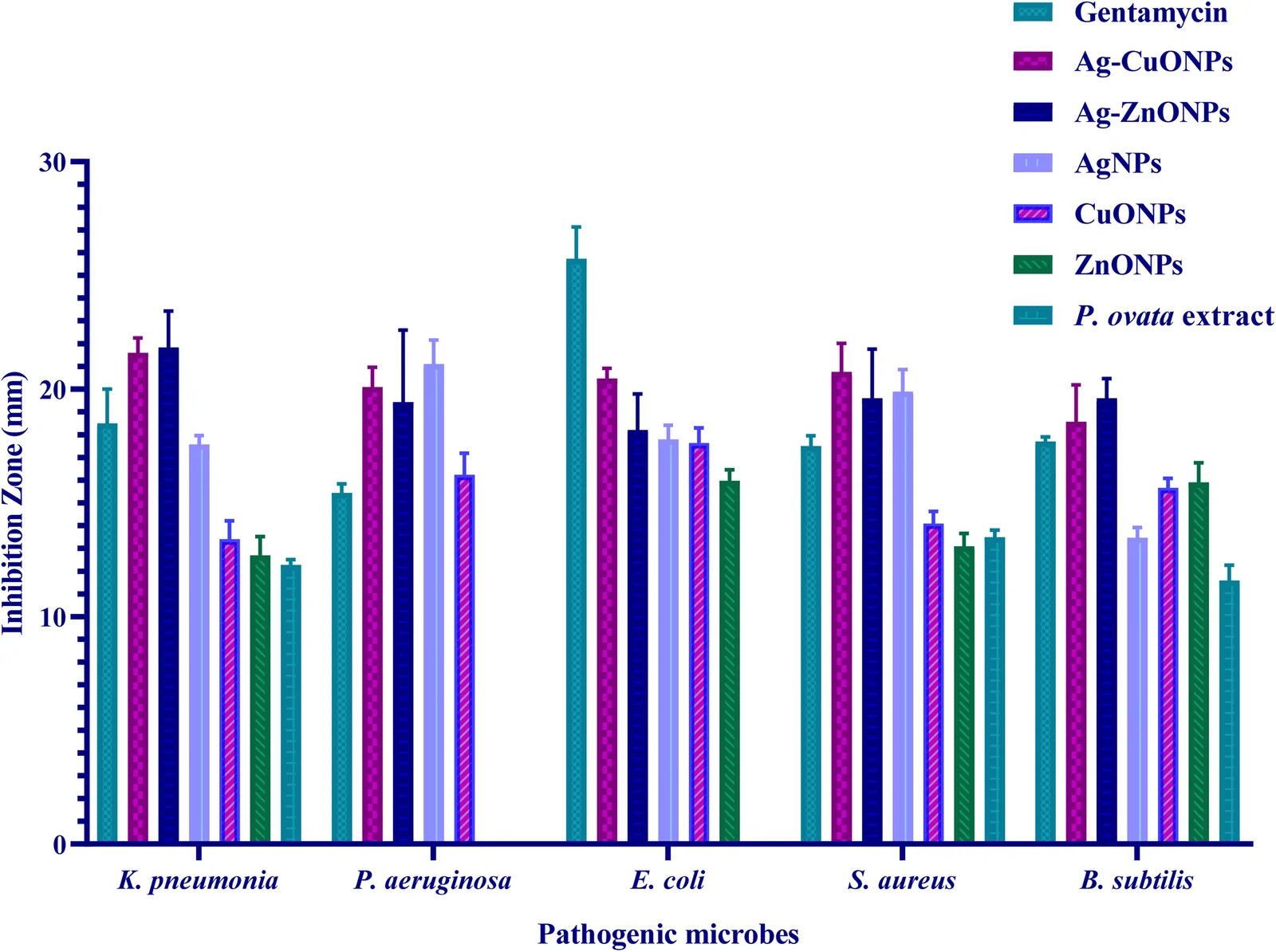
Antibacterial activity of the biosynthesized Ag–CuONPs and Ag–ZnONPs and their corresponding monometallic AgNPs, CuONPs, and ZnONPs against *Klebsiella pneumoniae*, *Pseudomonas aeruginosa*, *Escherichia coli*, *Staphylococcus aureus*, and *Bacillus subtilis*, as determined by the agar-well diffusion assay. Gentamicin and *Plantago ovata* extract were included as reference and comparator treatments, respectively. Antibacterial activity is expressed as the inhibition-zone diameter (mm). Data are presented as mean ± SD of three independent replicates (*n* = 3).

The antibacterial activities of Ag–CuONPs and Ag–ZnONPs were compared with those of AgNPs, CuONPs, ZnONPs, free *Plantago ovata* extract, metal-salt precursors, and gentamicin (Fig. 10). Ag–CuONPs produced inhibition zones of 21.60, 20.10, 20.47, 20.77, and 18.57 mm against *K. pneumoniae*, *P. aeruginosa*, *E. coli*, *S. aureus*, and *B. subtilis*, respectively. Ag–ZnONPs produced corresponding zones of 21.83, 19.43, 18.20, 19.60, and 19.60 mm. The broad-spectrum activity of Ag–CuONPs agrees with recent findings for biosynthesized Ag–CuO nanomaterials.^55^ Both mixed-phase formulations generally exhibited greater activity than CuONPs and ZnONPs. AgNP activity varied among the tested strains, with inhibition zones ranging from 13.47 to 21.10 mm. Ag–ZnONPs were most active against *K. pneumoniae*, whereas Ag–CuONPs showed greater activity against *P. aeruginosa*, *E. coli*, and *S. aureus*. Enhanced antibacterial performance has similarly been reported for green-synthesized Ag–ZnO nanomaterials.^56^ However, the observed improvement should not be described as definitive synergy because a physical mixture of the corresponding monometallic nanoparticles was not evaluated. The mixed-phase materials produced larger inhibition zones than gentamicin against most tested strains, whereas gentamicin was most active against *E. coli* at 25.73 mm. Such strain-dependent responses have also been reported for Ag-, CuO-, and mixed Ag–CuO nanoparticles.^57^ Nevertheless, inhibition-zone dimensions are affected by nanoparticle aggregation and diffusion through the agar and should therefore be interpreted together with broth-dilution measurements.^58^ Free *P. ovata* extract showed weak activity against *K. pneumoniae*, *S. aureus*, and *B. subtilis* and no detectable activity against *P. aeruginosa* or *E. coli*. Moreover, AgNO_3_, Cu(NO_3_)_2_, and Zn(NO_3_)_2_ produced no detectable inhibition zones under the applied conditions. The stronger activity of Ag–CuONPs and Ag–ZnONPs therefore could not be attributed solely to the free extract or unreacted salts. However, the absence of inhibition zones indicates only a lack of detectable activity under the specific concentrations and diffusion conditions employed.^59^ Ag–CuONPs exhibited MIC/MBC values of 125/250 µg mL^−1^ against *K. pneumoniae* and *P. aeruginosa*, 250/250 µg mL^−1^ against *E. coli*, 250/500 µg mL^−1^ against *S. aureus*, and 500/1000 µg mL^−1^ against *B. subtilis*. Ag–ZnONPs showed corresponding values of 250/250, 500/500, 500/1000, 250/500, and 250/500 µg mL^−1^. Thus, Ag–CuONPs generally showed greater broth-dilution activity, particularly against *P. aeruginosa* and *E. coli*. Differences between agar-diffusion and broth-dilution results are expected because these methods measure distinct aspects of antimicrobial performance.^48^ Collectively, the findings demonstrate that formation of the Ag–CuO and Ag–ZnO mixed-phase materials improved the consistency of antibacterial activity relative to most monometallic, extract, and precursor controls.

#### 3.4.2 Biofilm inhibition

**Fig. 11:**
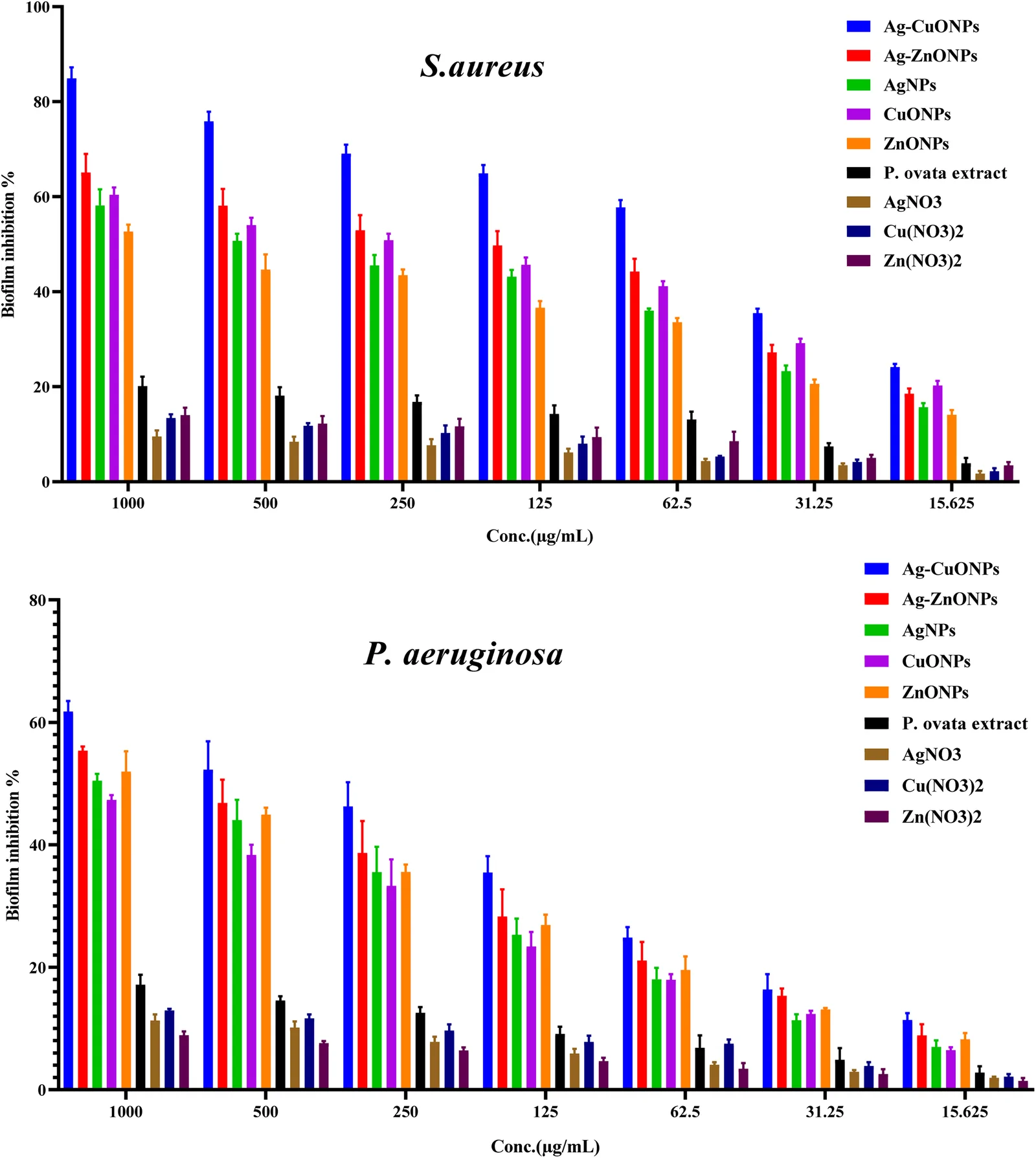
Concentration-dependent inhibition of biofilm formation by Ag–CuO and Ag–ZnO nanomaterials, their corresponding monometallic nanoparticles, free *Plantago ovata* extract, and metal-salt precursors against *Staphylococcus aureus* and *Pseudomonas aeruginosa*. Biofilm inhibition was determined using the crystal violet microtiter-plate assay. Data are presented as mean ± SD of three independent experiments (*n* = 3). Statistical differences among treatments at each concentration were evaluated separately for each bacterial strain using two-way ANOVA followed by Tukey's multiple-comparisons test.

The synthesized nanomaterials inhibited biofilm formation by *S. aureus* and *P. aeruginosa* in a concentration-dependent manner (Fig. 11). Against *S. aureus*, Ag–CuONPs exhibited the highest activity, reaching 84.91% inhibition at 1000 µg mL^−1^, followed by Ag–ZnONPs (65.08%), CuONPs (60.39%), AgNPs (58.15%), and ZnONPs (52.63%). Ag–CuONPs maintained appreciable activity at lower concentrations, producing 57.74% inhibition at 62.5 µg mL^−1^. Previous comparative investigations have similarly demonstrated stronger antibiofilm activity for Ag–CuO nanomaterials than their individual AgNPs and CuONPs components.^60^ Biofilm inhibition against *P. aeruginosa* was generally lower. At 1000 µg mL^−1^, Ag–CuONPs produced 61.78% inhibition, followed by Ag–ZnONPs (55.38%), ZnONPs (51.95%), AgNPs (50.46%), and CuONPs (47.33%). At 500 µg mL^−1^, Ag–CuONPs retained 52.27% inhibition, whereas the remaining formulations showed inhibition ranging from 38.36% to 46.84%. The lower susceptibility of *P. aeruginosa* may be associated with its outer membrane, extracellular polymeric matrix, efflux systems, and adaptable biofilm architecture. Nevertheless, Ag–ZnO nanomaterials have recently demonstrated effective inhibition of *P. aeruginosa* biofilm development and surface colonization.^61^ Both mixed-phase formulations produced greater inhibition than their corresponding monometallic comparators at the highest tested concentration. Their enhanced performance may reflect composition-dependent differences in particle–cell interactions, surface properties, aggregation, and metal-ion availability. AgNPs can interfere with bacterial adhesion, membrane integrity, and established biofilm structures,^62^ whereas ZnO-based nanoparticles have been reported to reduce biofilm biomass and disrupt its architecture.^63^ However, the present comparison does not establish synergy because an equivalent physical mixture of the corresponding monometallic nanoparticles was not tested. The free *P. ovata* extract showed substantially weaker activity, reaching only 20.15% and 17.15% inhibition against *S. aureus* and *P. aeruginosa*, respectively, at 1000 µg mL^−1^. The AgNO_3_, Cu(NO_3_)_2_, and Zn(NO_3_)_2_ precursor controls also exhibited limited inhibition compared with the synthesized nanomaterials. These findings indicate that the observed antibiofilm activity was associated mainly with the final nanomaterial formulations rather than the free extract or precursor salts. Nanomaterials may inhibit biofilm development through multiple processes, including reduced bacterial adhesion, membrane perturbation, interference with extracellular-polymeric-substance production, and suppression of biofilm-associated metabolic activity.^64^ Comparable concentration-dependent antibiofilm activity has been reported for biosynthesized Ag–ZnO materials.^65^ Nevertheless, these mechanisms were not investigated directly in the present study and should therefore be considered possible explanations rather than experimentally established pathways.

#### 3.4.3 Membrane disruption and modulation of biofilm-associated virulence genes

**Fig. 12:**
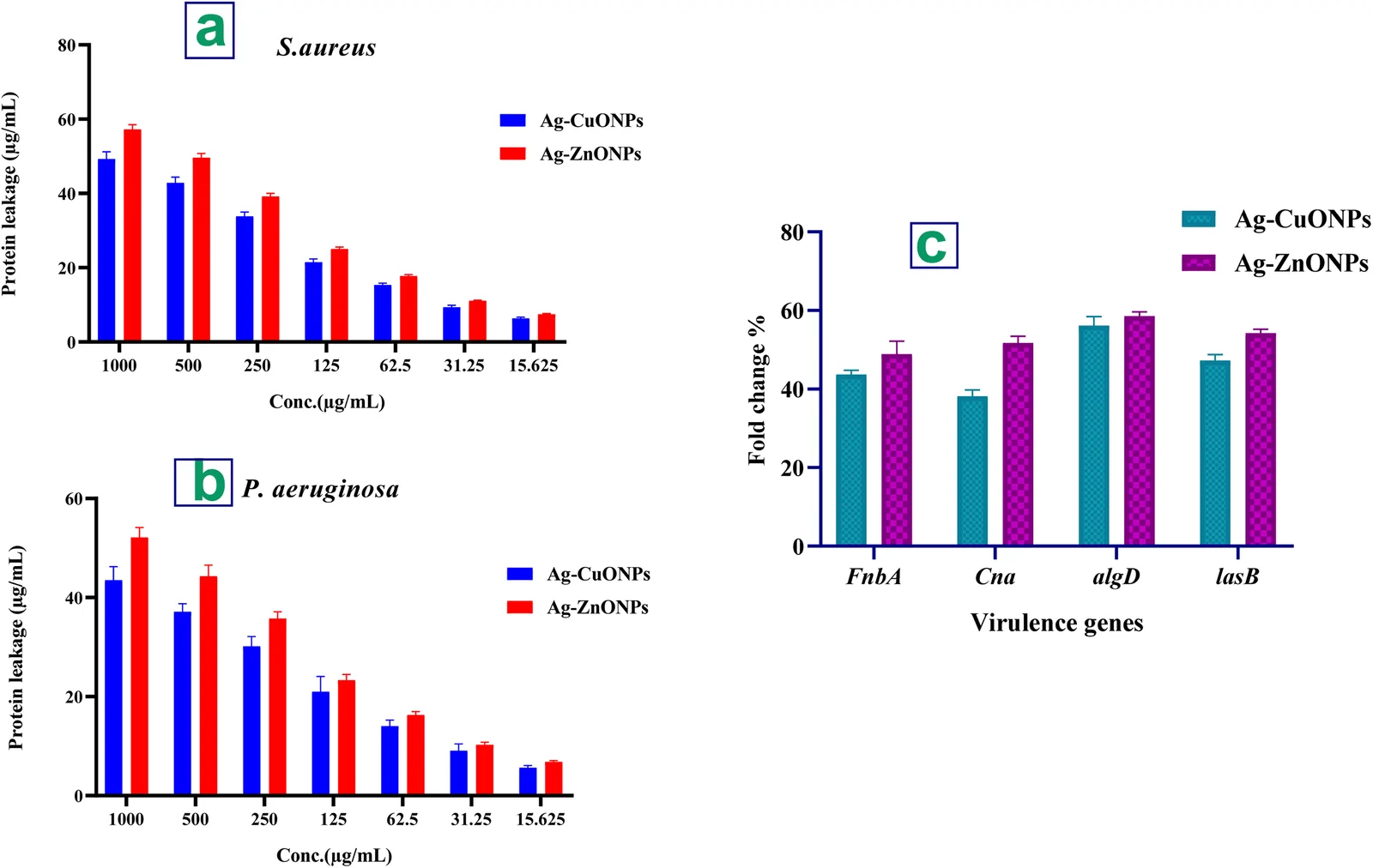
Effects of Ag–CuO and Ag–ZnO nanomaterials on membrane integrity and virulence-gene expression. Concentration-dependent extracellular protein leakage from (a) *Staphylococcus aureus* and (b) *Pseudomonas aeruginosa* following exposure to the tested nanomaterials. (c) Relative expression levels of the *S. aureus fnbA* and *cna* genes and the *P. aeruginosa algD* and *lasB* genes following treatment with Ag–CuO and Ag–ZnO. Gene-expression levels were normalized relative to the corresponding untreated controls. Data are presented as mean ± SD of three replicates (*n* = 3).

As shown in Fig. 12a and b, extracellular protein leakage was measured to assess changes in bacterial membrane permeability following exposure to Ag–CuONPs and Ag–ZnONPs. Both nanomaterials caused concentration-dependent protein leakage from *S. aureus* and *P. aeruginosa*. Increased extracellular protein concentration is commonly interpreted as evidence of impaired membrane integrity; however, it does not independently identify the molecular mechanism responsible for membrane damage.^66,67^ For Ag–CuONPs, protein leakage from *S. aureus* increased from 6.36 µg mL^−1^ at 15.625 µg mL^−1^ to 49.28 µg mL^−1^ at 1000 µg mL^−1^, whereas leakage from *P. aeruginosa* increased from 5.66 to 43.50 µg mL^−1^ over the same concentration range. Ag–ZnONPs produced a stronger response, increasing protein leakage from 7.48 to 57.26 µg mL^−1^ in *S. aureus* and from 6.82 to 52.15 µg mL^−1^ in *P. aeruginosa*. These findings indicate that Ag–ZnONPs caused greater concentration-dependent membrane permeabilization than Ag–CuONPs under the applied experimental conditions. Similar membrane-disruptive effects have been reported for metal and metal-oxide nanoparticles, where nanoparticle–surface interactions can alter membrane organization and promote leakage of intracellular constituents.^62^ Both formulations caused slightly greater protein leakage from *S. aureus* than from *P. aeruginosa* (Fig. 12a and b). This difference may reflect the distinct cell-envelope structures of Gram-positive and Gram-negative bacteria. The outer membrane of *P. aeruginosa*, together with its lipopolysaccharide layer, porins, and efflux systems, can restrict the penetration and cellular interaction of antimicrobial agents. Nevertheless, susceptibility to nanoparticles also depends on particle composition, size, surface charge, aggregation, and exposure conditions; therefore, the difference between the two strains should not be attributed solely to Gram classification.^63^

The nanomaterials also reduced the expression of biofilm- and virulence-associated genes (Fig. 12c). In *S. aureus*, Ag–CuONPs decreased *fnbA* and *cna* expression by 43.67% and 38.19%, respectively, whereas Ag–ZnONPs produced reductions of 48.91% and 51.71%. The *fnbA* and *cna* genes encode fibronectin- and collagen-binding adhesins, respectively, which contribute to bacterial attachment, colonization, and biofilm establishment. Suppression of these genes may therefore impair the initial adhesion and subsequent maturation of *S. aureus* biofilms. Comparable downregulation of *fnbA* and *cna* has been reported following exposure of *S. aureus* to biosynthesized AgNPs and ZnO-based nanoparticles.^68,69^ In *P. aeruginosa*, Ag–CuONPs reduced the expression of *algD* and *lasB* by 56.17% and 47.23%, respectively, while Ag–ZnONPs caused reductions of 58.55% and 54.26% (Fig. 12c). The *algD* gene is involved in alginate biosynthesis and contributes to the formation and structural stability of the biofilm matrix, whereas *lasB* encodes LasB elastase, an important quorum-sensing-regulated virulence factor. Their reduced expression suggests that both mixed-phase nanomaterials may interfere with biofilm-matrix production and virulence-factor regulation. Nanoparticle-mediated suppression of *P. aeruginosa* biofilm and virulence genes, including *lasB* and genes associated with extracellular-matrix production, has similarly been reported.^61,70^ Collectively, Ag–ZnONPs produced greater protein leakage and stronger suppression of all four investigated genes than Ag–CuONPs. The agreement between membrane-permeability changes and transcriptional suppression supports a multifactorial antibacterial response involving membrane damage and attenuation of biofilm-associated functions. Nevertheless, these findings do not demonstrate a direct causal relationship between protein leakage and altered gene expression, nor do they establish a specific mechanism involving reactive oxygen species or metal-ion release because these parameters were not measured directly.

### 3.5 Cellular metabolic and anticancer effects

#### 3.5.1 Vero-cell metabolic activity

**Fig. 13:**
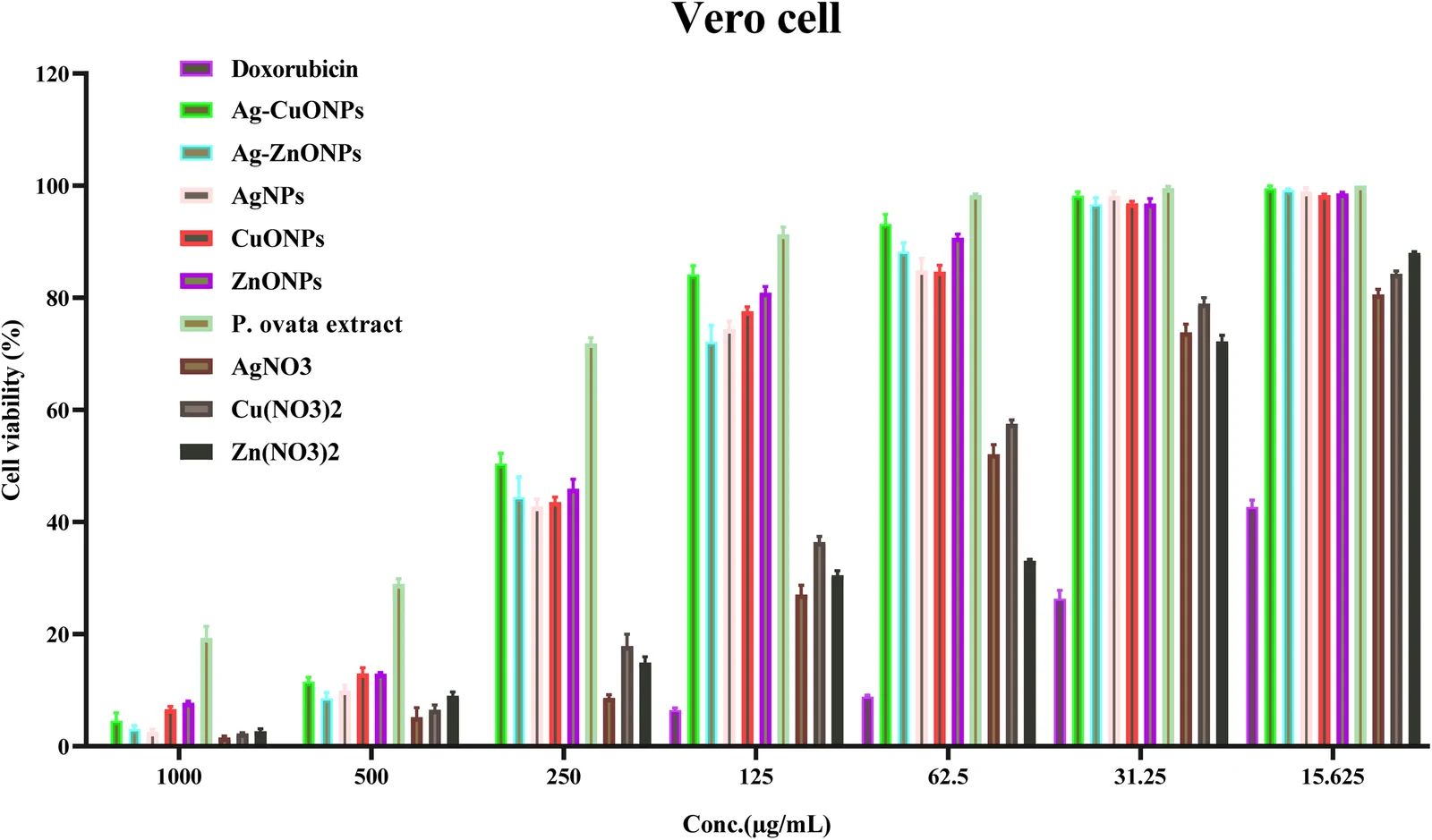
Concentration-dependent effects of Ag–CuONPs and Ag–ZnONPs, their corresponding monometallic nanoparticles, free *Plantago ovata* extract, metal-salt precursors, and doxorubicin on the metabolic activity of Vero cells. Cellular metabolic activity was determined using the MTT assay and expressed relative to the untreated control, which was set at 100%. Data are presented as mean ± SD of three replicates (*n* = 3). The effects of treatment and concentration were analysed using two-way ANOVA followed by Tukey's multiple-comparisons test.

The effects of the synthesized nanomaterials and comparator treatments on Vero-cell metabolic activity are shown in Fig. 13. After 48 h, all nanoparticle formulations produced concentration-dependent reductions in metabolic activity. At 62.5 µg mL^−1^, the values remained relatively high at 93.17, 88.25, 84.91, 84.69, and 90.73% for Ag–CuONPs, Ag–ZnONPs, AgNPs, CuONPs, and ZnONPs, respectively. At 125 µg mL^−1^, the corresponding values decreased to 84.16, 72.11, 74.44, 77.61, and 80.94%, while at 250 µg mL^−1^ they reached 50.41, 44.44, 42.73, 43.55, and 45.95%, respectively. At 1000 µg mL^−1^, metabolic activity was reduced to 4.56–7.76%. Similar concentration-dependent responses have been reported for green-synthesized ZnO nanoparticles in Vero cells,^71^ Ag and ZnO nanoparticles in mammalian cells,^72^ and plant-derived metal-oxide nanocomposites.^73^ The IC_50_ values were 253.75 ± 9.80, 225.17 ± 14.96, 221.49 ± 3.33, 226.34 ± 3.01, and 235.77 ± 4.84 µg mL^−1^ for Ag–CuONPs, Ag–ZnONPs, AgNPs, CuONPs, and ZnONPs, respectively. Ag–CuONPs therefore exhibited the numerically highest IC_50_ among the nanoparticle formulations. However, the values were relatively close, and no significant difference should be claimed unless confirmed by the two-way ANOVA and Tukey multiple-comparisons analysis. Differences in cellular responses to metal and metal-oxide nanoparticles may depend on their composition, size, aggregation, surface properties, cellular uptake, and dissolution behaviour.^74^ The free *Plantago ovata* extract exhibited the weakest effect, with an IC_50_ of 377.34 ± 5.67 µg mL^−1^. In contrast, AgNO_3_, Cu(NO_3_)_2_, and Zn(NO_3_)_2_ showed substantially lower IC_50_ values of 67.85 ± 4.06, 84.80 ± 2.36, and 48.98 ± 0.50 µg mL^−1^, respectively. These results demonstrate that the final nanoparticle effects were not attributable solely to the free extract and that the metal-salt precursors exerted stronger effects under the tested conditions. Nevertheless, because the salts and nanoparticles were compared at equal nominal mass concentrations rather than equivalent elemental-metal concentrations, these results cannot be used to quantify metal-ion release or bioavailability. Doxorubicin produced the strongest response and reduced metabolic activity below 50% at the lowest tested concentration; therefore, its IC_50_ was reported as <15.625 µg mL^−1^. Overall, the nanoparticles produced relatively limited changes in Vero-cell metabolic activity at concentrations up to 62.5 µg mL^−1^ but substantial suppression at higher concentrations. Selectivity should be evaluated by comparing these Vero-cell IC_50_ values with those obtained for Caco-2 and MCF-7 cancer cells. Moreover, because MTT measures cellular metabolic reduction rather than cell death directly and nanoparticles may interfere with tetrazolium-based assays, these findings should be interpreted together with cellular morphology and complementary biological assays.^75^

#### 3.5.2 Caco-2 and MCF-7 metabolic activity

**Fig. 14:**
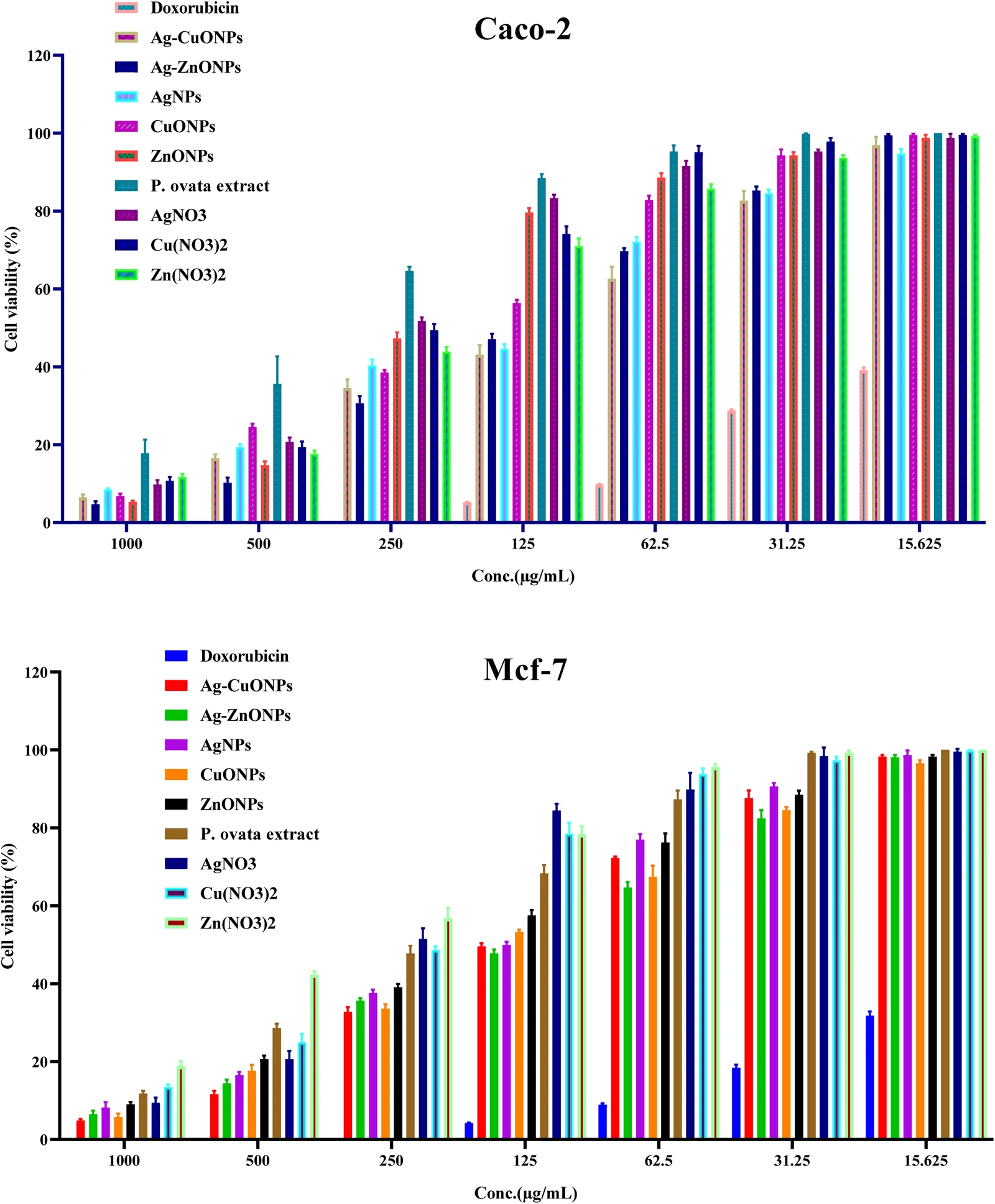
Concentration-dependent effects of Ag–CuO and Ag–ZnO nanomaterials, their corresponding monometallic nanoparticles, free *Plantago ovata* extract, metal-salt precursors, and doxorubicin on the metabolic activity of Caco-2 colorectal cancer cells and MCF-7 breast cancer cells. Cell viability was determined using the MTT assay and normalized relative to the untreated control, which was considered 100%. Data are presented as mean ± SD of three replicates (*n* = 3). Statistical differences among treatments at each concentration were evaluated separately for each cell line using two-way ANOVA followed by Tukey's multiple-comparisons test.

The synthesized nanomaterials caused concentration-dependent reductions in the metabolic activity of both Caco-2 and MCF-7 cancer cells after 48 h of exposure (Fig. 14). At the highest concentration of 1000 µg mL^−1^, the metabolic activity of Caco-2 cells decreased to approximately 2.5–7.8% following treatment with the different nanoparticle formulations. Similarly, MCF-7 metabolic activity was reduced to approximately 4.9–9.0%. This concentration-dependent response is consistent with recent observations that the biological effects of metallic and metal-oxide nanoparticles are strongly influenced by exposure concentration, particle composition, and cancer-cell type.^76^ In Caco-2 cells, Ag–CuONPs exhibited the greatest activity among the nanoparticle formulations, with an IC_50_ of 102.89 ± 8.05 µg mL^−1^. AgNPs and Ag–ZnONPs showed comparable IC_50_ values of 112.93 ± 1.76 and 117.14 ± 3.47 µg mL^−1^, respectively. By comparison, CuONPs and ZnONPs were less active, with IC_50_ values of 169.81 ± 4.99 and 382.37 ± 34.27 µg mL^−1^, respectively. At 125 µg mL^−1^, the residual metabolic activities were approximately 43.15% for Ag–CuONPs, 44.64% for AgNPs, 47.14% for Ag–ZnONPs, 56.40% for CuONPs, and 79.65% for ZnONPs. The relatively pronounced response of Caco-2 cells to Ag-containing materials agrees with recent findings demonstrating concentration-dependent inhibition of Caco-2 cells by green-synthesized AgNPs.^77^ The greater activity of Ag–CuONPs than CuONPs against Caco-2 cells suggests that the combined material possessed a biological response different from that of the corresponding monometallic CuO formulation. This difference may be associated with changes in particle size, surface properties, cellular interaction, aggregation state, or the availability of Ag- and Cu-containing species. However, it should not be described as synergistic because synergy was not evaluated using an equivalent physical mixture or a formal combination model. Recent studies of green-synthesized CuO-containing nanomaterials have similarly demonstrated that their anticancer effects depend on physicochemical properties and the tested cellular model.^73^ In MCF-7 cells, Ag–ZnONPs showed the lowest nanoparticle IC_50_ value of 116.98 ± 3.23 µg mL^−1^, followed by Ag–CuONPs and AgNPs at 124.35 ± 2.51 and 127.03 ± 5.19 µg mL^−1^, respectively. The corresponding IC_50_ values of CuONPs and ZnONPs were 145.91 ± 3.06 and 176.14 ± 6.58 µg mL^−1^. At 125 µg mL^−1^, the metabolic activities of MCF-7 cells were approximately 47.83% for Ag–ZnONPs, 49.63% for Ag–CuONPs, 50.00% for AgNPs, 53.30% for CuONPs, and 57.59% for ZnONPs. Thus, the Ag-containing mixed-phase formulations showed slightly greater activity than their corresponding monometallic metal oxides. Changes in the cellular response of Ag-modified ZnO relative to monometallic Ag and ZnO nanoparticles have also been reported in plant-mediated systems.^78^ The observed MCF-7 response is consistent with a recent meta-analysis showing that the effects of AgNPs and ZnONPs on MCF-7 cells vary considerably with nanoparticle concentration, exposure duration, particle size, and synthesis route.^79^ Furthermore, green-synthesized CuONPs have recently been shown to reduce the metabolic activity of MCF-7 and colorectal cancer cells in a concentration-dependent manner.^80^ Therefore, differences among the tested formulations cannot be attributed solely to their nominal chemical identities and may also reflect differences in hydrodynamic size, polydispersity, surface charge, aggregation, and interactions with components of the culture medium. The free *Plantago ovata* extract was less active than Ag–CuONPs and Ag–ZnONPs, producing IC_50_ values of 239.85 ± 5.33 µg mL^−1^ against Caco-2 cells and 236.72 ± 12.17 µg mL^−1^ against MCF-7 cells. The metal-salt precursors also produced higher IC_50_ values against the cancer cells than the target mixed-phase nanomaterials. These comparisons indicate that the biological activity of Ag–CuONPs and Ag–ZnONPs cannot be explained solely by the free extract or residual precursor salts. Nevertheless, comparisons among nanoparticles, extract, and salts were based on equal nominal mass concentrations rather than equivalent molar or elemental-metal concentrations and should therefore be interpreted qualitatively. Doxorubicin was the most active treatment against both cancer-cell lines, with an IC_50_ below the lowest tested concentration of 15.625 µg mL^−1^. Comparison with the Vero-cell results showed approximate selectivity indices of 2.47 and 2.04 for Ag–CuONPs against Caco-2 and MCF-7 cells, respectively. Ag–ZnONPs produced corresponding selectivity indices of 1.92 and 1.93, while those of AgNPs were 1.96 and 1.74. Ag–CuONPs therefore showed the greatest preferential activity toward the malignant cells among the nanoparticle formulations. However, these values indicate only moderate selectivity and do not establish tumour-specific activity. The marked dependence of nanoparticle effects on cellular phenotype and exposure conditions has been emphasized in recent evaluations of nanoparticle–cell interactions.^75^

#### 3.5.3 Apoptosis and cell-cycle distribution

**Fig. 15:**
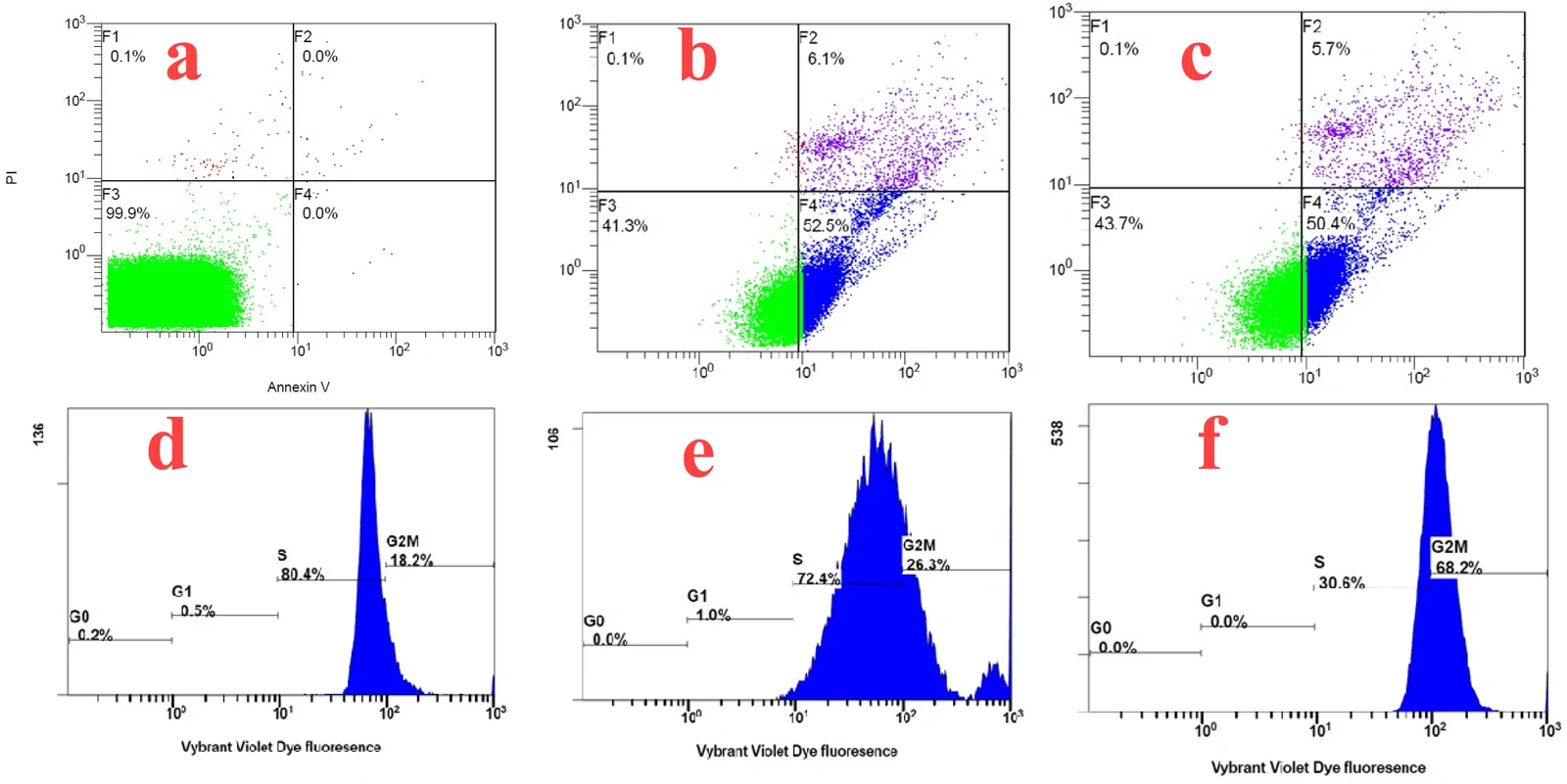
Flow cytometry analysis of apoptosis and cell cycle distribution in Caco-2 cells following treatment with *Plantago ovata*-mediated nanoparticles. Annexin V/PI dot plots showing apoptotic cell populations for (a) control, (b) Ag–CuONPs, and (c) Ag–ZnONPs. Cell cycle distribution histograms illustrating the percentage of cells in different phases for (d) control, (e) Ag–CuONPs, and (f) Ag–ZnONPs.

Annexin V/PI flow-cytometric analysis demonstrated a pronounced increase in apoptotic cell populations following treatment of Caco-2 cells with Ag–CuONPs and Ag–ZnONPs (Fig. 15a–c). Untreated cells showed 99.9% viability, whereas the viable population decreased to 41.3% following Ag–CuONPs treatment. According to the quadrant classification applied during flow-cytometric analysis, Ag–CuONPs treatment resulted in 52.5% early apoptotic cells, 0.1% late apoptotic cells, and 6.1% necrotic cells. Ag–ZnONPs produced a comparable response, reducing the viable population to 43.7%, with early apoptotic, late apoptotic, and necrotic populations of 50.4%, 0.1%, and 5.7%, respectively. In this analysis, F3, F4, F1, and F2 represented viable, early apoptotic, late apoptotic, and necrotic cells, respectively. Annexin V binding indicates phosphatidylserine externalization, whereas PI uptake reflects loss of plasma-membrane integrity; however, interpretation of the individual populations was based on the quadrant classification defined in the applied analytical protocol.^81^ These findings indicate that apoptosis was the predominant form of treatment-associated cell death under the applied conditions. The differences between Ag–CuONPs and Ag–ZnONPs were relatively small; therefore, Ag–CuONPs should only be described as more effective if this difference was statistically significant. Similar increases in Annexin V-positive populations have been reported following treatment with Ag/CuO-containing nanomaterials.^82^ Nevertheless, Annexin V/PI analysis alone cannot establish whether apoptosis proceeded through the intrinsic or extrinsic pathway. Cell-cycle analysis showed different responses to the two bimetallic formulations (Fig. 15d–f). Untreated cells were distributed predominantly in S phase and G_2_/M phase, representing approximately 58.4 and 38.2% of the gated population, respectively. Following Ag–CuONPs treatment, the corresponding populations were 57.2% in S phase and 28.5% in G_2_/M. Because the S-phase fraction did not increase relative to the untreated control, these data do not provide clear evidence of S-phase arrest. Instead, the Ag–CuONPs response should be described as an alteration of cell-cycle distribution accompanying extensive apoptosis. In contrast, Ag–ZnONPs treatment increased the G_2_/M population from 38.2 to 68.2%, together with a reduction in the S-phase population to 30.6%. This marked redistribution supports G_2_/M-phase accumulation and suggests inhibition of progression through the G_2_/M checkpoint. G_2_/M accumulation has also been reported for ZnO-containing nanomaterials and may occur when cellular stress prevents completion of DNA replication or mitotic progression.^83^ However, confirmation of the responsible molecular pathway would require analysis of relevant checkpoint regulators, such as cyclin B1, CDK1, p21, p53, or phosphorylated histone H3. Inclusive, both Ag–CuONPs and Ag–ZnONPs induced substantial apoptosis in Caco-2 cells, whereas a distinct G_2_/M-phase accumulation was primarily observed following Ag–ZnONPs treatment. These results extend the MTT findings by showing that the reduction in cellular metabolic activity was accompanied by an increase in Annexin V-positive cell populations. Nevertheless, the present data do not independently establish oxidative stress, mitochondrial dysfunction, or a specific apoptotic pathway.

#### 3.5.4 Apoptosis-associated gene expression

**Fig. 16:**
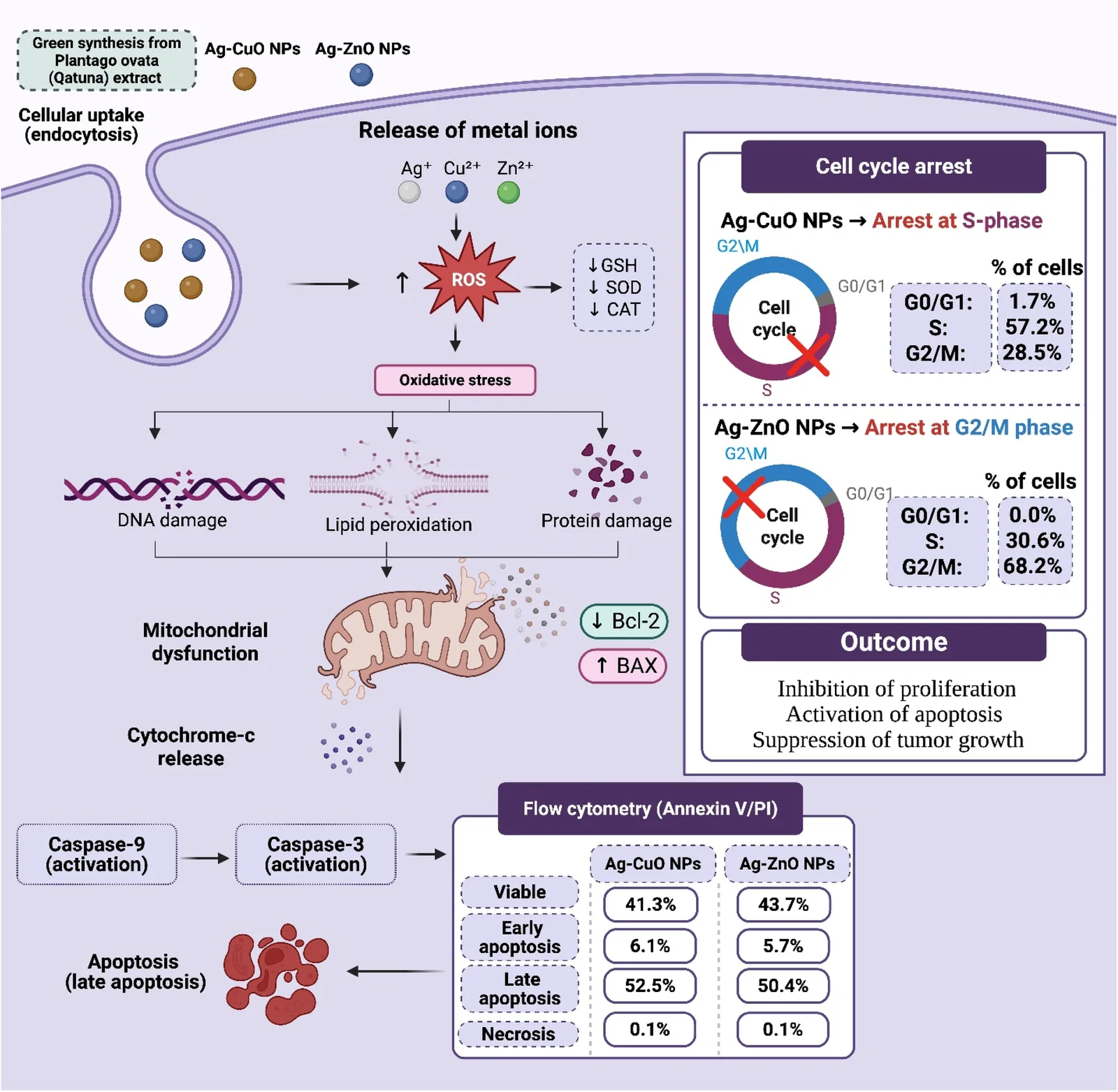
Schematic representation of the experimentally observed and proposed apoptosis-associated effects of *Plantago ovata*-mediated Ag–CuONPs and Ag–ZnONPs in Caco-2 cells. Annexin V/PI flow cytometry demonstrated increased apoptotic populations following treatment with both nanomaterials, while cell-cycle analysis showed marked G_2_/M-phase accumulation following Ag–ZnONPs treatment. The depicted cellular uptake, metal-ion release, ROS generation, antioxidant-enzyme depletion, macromolecular damage, mitochondrial dysfunction, cytochrome-*c* release, and caspase activation represent literature-supported proposed events and were not directly measured in the present study.

Treatment with Ag–CuONPs and Ag–ZnONPs altered the transcriptional expression of the apoptosis-associated genes *BAX*, *BCL2*, and *CASP3* in Caco-2 cells (Table 2). Relative to the untreated control, Ag–CuONPs increased *CASP3* and *BAX* expression to 3.85 ± 0.21- and 3.20 ± 0.18-fold, respectively, while reducing *BCL2* expression to 0.42 ± 0.03-fold. Ag–ZnONPs similarly increased *CASP3* and *BAX* expression to 3.10 ± 0.17- and 2.75 ± 0.15-fold, respectively, and decreased *BCL2* expression to 0.55 ± 0.04-fold. The calculated *BAX*/*BCL2* expression ratios increased from approximately 1.0 in untreated cells to 7.62 following Ag–CuONPs treatment and 5.00 following Ag–ZnONPs treatment. The shift toward higher *BAX* and lower *BCL2* expression indicates a pro-apoptotic transcriptional balance. *BAX* promotes mitochondrial outer-membrane permeabilization, whereas *BCL-2* opposes this process; consequently, the balance between these proteins contributes to the regulation of mitochondrial apoptosis.^84^ Comparable upregulation of *BAX* and *CASP3*, together with suppression of *BCL2*, has been reported following exposure of cancer cells to biosynthesized Ag–CuO nanoparticles.^82^ The increase in *CASP3* transcription was also consistent with the elevated apoptotic populations detected by Annexin V/PI analysis. Similar changes in *BAX*, *BCL2*, and caspase-associated genes have been reported for Ag-modified ZnO nanoparticles.^78^ However, increased *CASP3* mRNA expression does not necessarily demonstrate proteolytic activation of caspase-3. Confirmation would require measurement of cleaved caspase-3 or caspase-3/7 enzymatic activity. Likewise, confirmation of mitochondrial involvement would require assessment of mitochondrial membrane potential, cytochrome-*c* release, caspase-9 activation, or related mitochondrial markers.^85^ Taken together, the Annexin V/PI and gene-expression findings support apoptosis induction and a shift toward a pro-apoptotic molecular profile following treatment with both bimetallic formulations. Ag–CuONPs produced the greater *BAX*/*BCL2* expression ratio, whereas Ag–ZnONPs produced the clearer G_2_/M-phase accumulation. These complementary responses suggest composition-dependent effects on Caco-2 cell fate. Nevertheless, because intracellular ROS, mitochondrial membrane potential, cytochrome-c release, and caspase activity were not directly measured, the findings should not be described as definitive evidence of an ROS-driven mitochondrial pathway.^86^ The experimentally observed findings and the proposed apoptosis-associated events in Ag–CuONPs- and Ag–ZnONPs-treated Caco-2 cells are summarized schematically in Fig. 16. The pathways involving metal-ion release, oxidative stress, mitochondrial dysfunction, and caspase activation are literature-supported hypotheses and were not directly examined in the present study.

**Table 2:** Relative expression levels of apoptosis-associated genes in Caco-2 cells following treatment with Ag–CuONPs and Ag–ZnONPs. Results are presented as mean ± SD of three independent experiments

Treatment	Caspase-3 (fold change)	*BAX* (fold change)	*Bcl-2* (fold change)
Control	1.00 ± 0.05	1.00 ± 0.04	1.00 ± 0.06
Ag–CuONPs	3.85 ± 0.21	3.20 ± 0.18	0.42 ± 0.03
Ag–ZnONPs	3.10 ± 0.17	2.75 ± 0.15	0.55 ± 0.04

### 3.6 DPPH and ABTS radical-scavenging activities

**Fig. 17:**
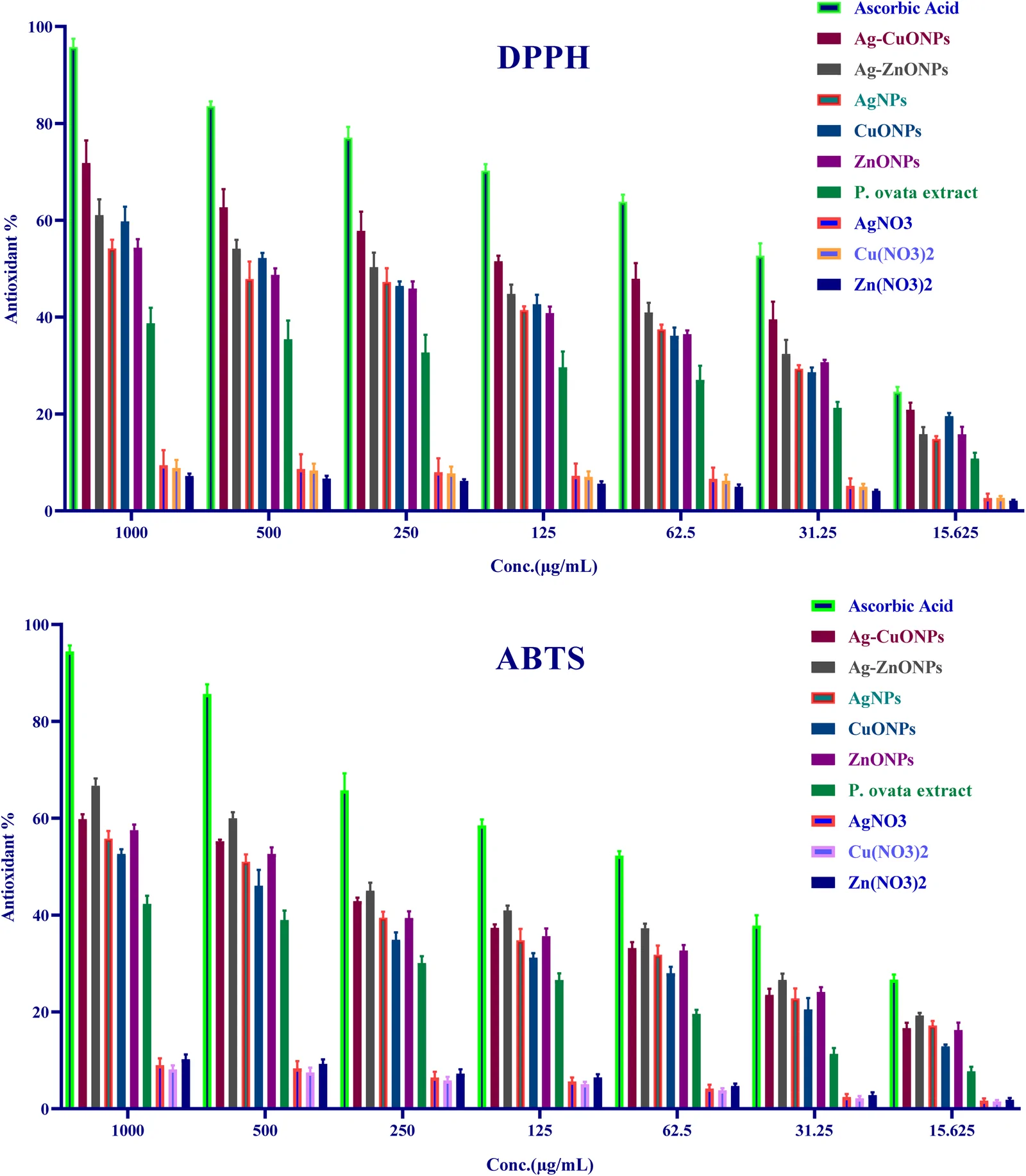
Concentration-dependent radical-scavenging activities of Ag–CuO and Ag–ZnO nanomaterials, their corresponding monometallic nanoparticles, free *Plantago ovata* extract, and metal-salt precursors, as determined using the DPPH and ABTS assays. Ascorbic acid was used as the positive control. Data are presented as mean ± SD of three independent experiments (*n* = 3). Statistical differences among treatments at each concentration were evaluated separately for each assay using two-way ANOVA followed by Tukey's multiple-comparisons test.

The synthesized nanomaterials exhibited concentration-dependent radical-scavenging activity in both assays (Fig. 17). At 1000 µg mL^−1^, ascorbic acid produced the highest DPPH inhibition (95.74%), followed by Ag–CuONPs (71.84%), Ag–ZnONPs (61.06%), CuONPs (59.77%), ZnONPs (54.34%), and AgNPs (54.19%). Concentration-dependent DPPH scavenging has similarly been reported for green-synthesized Ag–CuO nanomaterials.^87^ The DPPH IC_50_ values were 29.86 ± 1.24, 93.56 ± 33.77, 268.91 ± 102.41, 406.32 ± 28.46, 584.86 ± 94.16, and 590.75 ± 144.68 µg mL^−1^ for ascorbic acid, Ag–CuONPs, Ag–ZnONPs, CuONPs, ZnONPs, and AgNPs, respectively. Ag–CuONPs therefore showed the strongest DPPH-scavenging activity among the nanomaterials. Their improved response compared with the corresponding monometallic materials may reflect modification of the surface chemical environment and availability of redox-active sites following combined synthesis. However, this finding should not be described as synergy without comparison with an equivalent physical mixture. In the ABTS assay, inhibition at 1000 µg mL^−1^ reached 94.41% for ascorbic acid, 66.73% for Ag–ZnONPs, 59.81% for Ag–CuONPs, 57.55% for ZnONPs, 55.76% for AgNPs, and 52.65% for CuONPs. The corresponding IC_50_ values were 57.68 ± 0.95, 332.51 ± 24.07, 393.93 ± 8.63, 451.74 ± 21.98, 496.13 ± 58.93, and 773.14 ± 86.94 µg mL^−1^, respectively. Thus, Ag–ZnONPs were the most active nanomaterial in the ABTS assay. Enhanced radical-scavenging capacity following the incorporation of Ag into ZnO-based materials has also been reported previously,^88^ while antioxidant responses have been independently demonstrated for green-synthesized ZnONPs^89^ and AgNPs.^90^ The free *P. ovata* extract showed lower inhibition, reaching 38.72% in DPPH and 42.29% in ABTS, with IC_50_ values exceeding 1000 µg mL^−1^. The three metal-salt precursors produced only approximately 7–10% inhibition at 1000 µg mL^−1^ and also had IC_50_ values above 1000 µg mL^−1^. These controls indicate that the radical-scavenging activities of the synthesized nanomaterials cannot be attributed solely to the free extract or residual precursor salts. Comparable differences between plant extracts, metal precursors, and biosynthesized metal-oxide nanomaterials have been reported.^91^ The different activity rankings observed between DPPH and ABTS may reflect differences in radical structure, reaction medium, electron- and hydrogen-transfer behaviour, particle dispersibility, and accessibility of surface-active sites. Accordingly, these cell-free assays demonstrate chemical radical-scavenging capacity under the applied conditions but do not independently establish intracellular antioxidant protection. Furthermore, the relatively large SD values of some DPPH IC_50_ estimates, particularly those of Ag–ZnONPs and AgNPs, indicate replicate variability and warrant cautious interpretation of small differences between formulations.

### 3.7 Study limitations

The present study used a single *Plantago ovata* extract volume, and systematic optimization of extract concentration was not performed. Although three independent extract batches were standardized using pH, dry-solids concentration, and extraction yield, HPLC profiling and contaminant screening were not conducted separately for every batch. The extract was also not compared with conventional stabilizing agents or composition-matched nanomaterials produced by conventional synthesis. Washed-versus-unwashed formulations and equivalent physical mixtures were not evaluated; therefore, contributions from residual surface-associated species cannot be excluded, and the observed enhancement should not be interpreted as synergism. In addition, the absence of XPS, ICP, and ion-release analyses limited determination of oxidation states, bulk composition, and metal availability. Finally, the biological findings were based on *in vitro* models. Annexin V/PI and gene-expression results support an apoptosis-associated response but do not confirm mitochondrial involvement or functional caspase activation. Likewise, DPPH and ABTS demonstrate cell-free radical-scavenging capacity but not intracellular antioxidant protection. Further protein-level, ROS, stability, and *in vivo* studies are therefore required.

## 4 Conclusion

*Plantago ovata* seed extract was successfully employed as a plant-derived medium for the green synthesis of Ag–CuO and Ag–ZnO mixed-phase nanomaterials, while the corresponding monometallic nanoparticles served as comparative materials. Complementary characterization using UV-vis, FTIR, XRD, electron microscopy, HR-TEM, EDX, elemental mapping, DLS, zeta-potential analysis, and BET confirmed their optical, structural, morphological, elemental, colloidal, and textural characteristics. The extract-standardization results also demonstrated acceptable reproducibility among independently prepared batches. Both mixed-phase formulations exhibited broad-spectrum antibacterial, antibiofilm, radical-scavenging, and anticancer activities. Ag–CuONPs generally showed stronger antibacterial, antibiofilm, DPPH-scavenging, and Caco-2-directed effects, whereas Ag–ZnONPs demonstrated greater ABTS-scavenging activity and a comparatively stronger response against MCF-7 cells. Both formulations altered bacterial membrane permeability and reduced the expression of selected biofilm- and virulence-associated genes. They also induced substantial apoptosis in Caco-2 cells, accompanied by pro-apoptotic transcriptional changes, while Ag–ZnONPs produced a distinct accumulation of cells in the G_2_/M phase. Comparisons with the monometallic nanoparticles, free plant extract, and precursor salts indicated that the biological performance was associated mainly with the final mixed-phase formulations. However, the findings should not be interpreted as definitive evidence of synergistic activity because equivalent physical mixtures were not tested. Moreover, the precise contributions of metal-ion release, intracellular oxidative stress, and interfacial interactions were not directly determined. Further studies involving physicochemical stability in biological media, ion-release measurements, detailed molecular investigations, and *in vivo* evaluation are therefore required before biomedical application can be considered.

## Author contributions

Hayfa Habes Almutairi: resources, software, visualization, project administration, funding acquisition. Nada H. Aljarba: data curation, writing – original draft, investigation, project administration, funding acquisition. Sahar M. AlMotwaa: methodology, validation, visualization. Waad A. Al-Otaibi: writing – review and editing, formal analysis, visualization. Mohamed K. Y. Soliman: supervision, investigation, software, validation, conceptualization.

## Conflicts of interest

The authors declare no conflict of interest.

## Supplementary Material

RA-OLF-D6RA05100F-s001

RA-OLF-D6RA05100F-s002

## Data Availability

The datasets used and/or analyzed during the current study are available from the corresponding author on reasonable request. Supplementary information (SI) is available. See DOI: https://doi.org/10.1039/d6ra05100f.
