# Supplementary material for: Multifunctional bimetallic Ag–CuO and Ag–ZnO nanoparticles biosynthesized using Plantago ovata for biomedical applications
Source: RSC Adv. 2026 Sep 25;16(45):57574–602. doi: 10.1039/d6ra05100f (PMC13613714; doi:10.1039/d6ra05100f)
Supplement: Supplementary file 1 [file RA-016-D6RA05100F-s002.pdf]

Supplementary Information (SI) for RSC Advances.
This journal is © The Royal Society of Chemistry

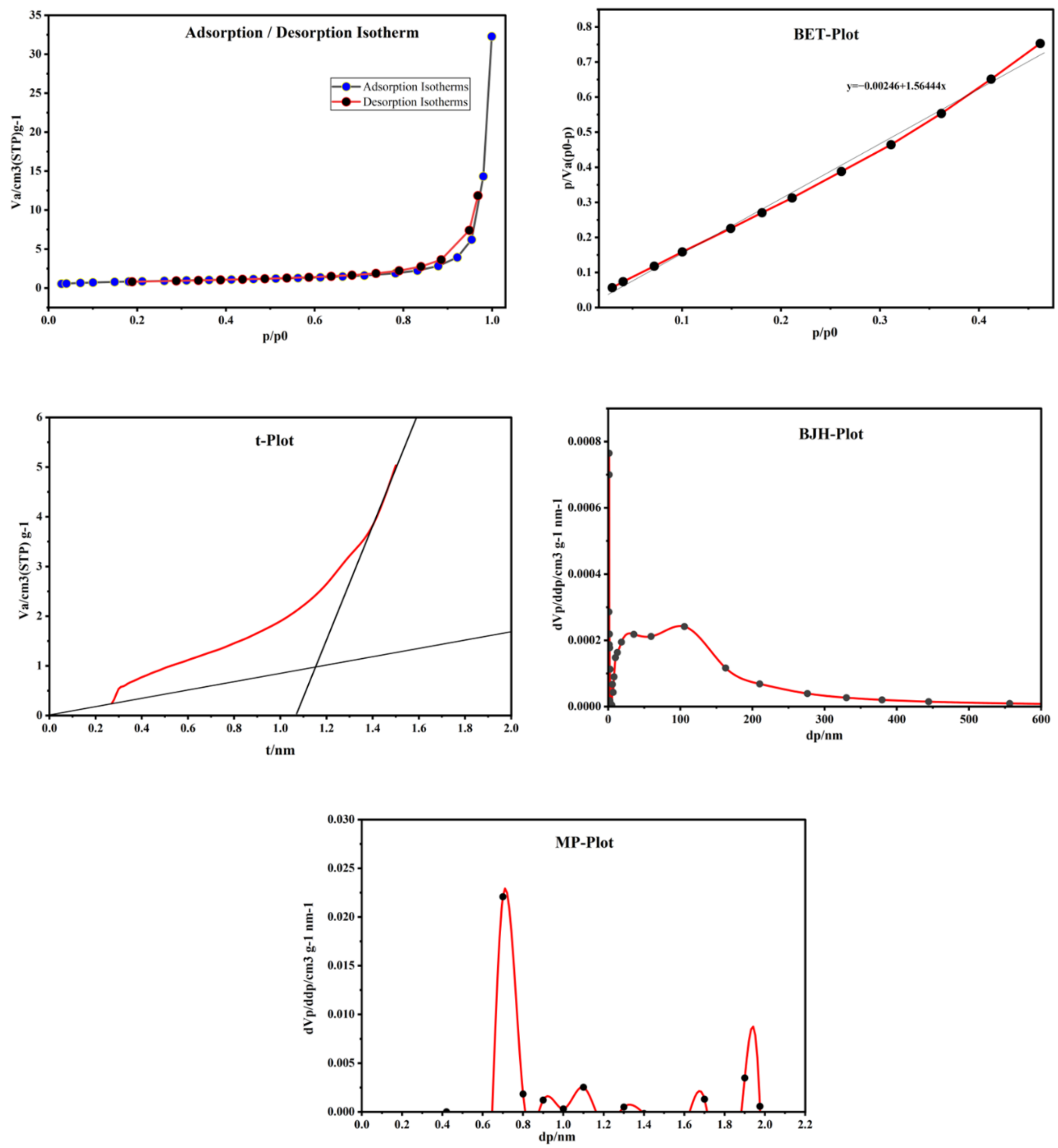

Figure S1. The BET analysis of Ag-CuONPs

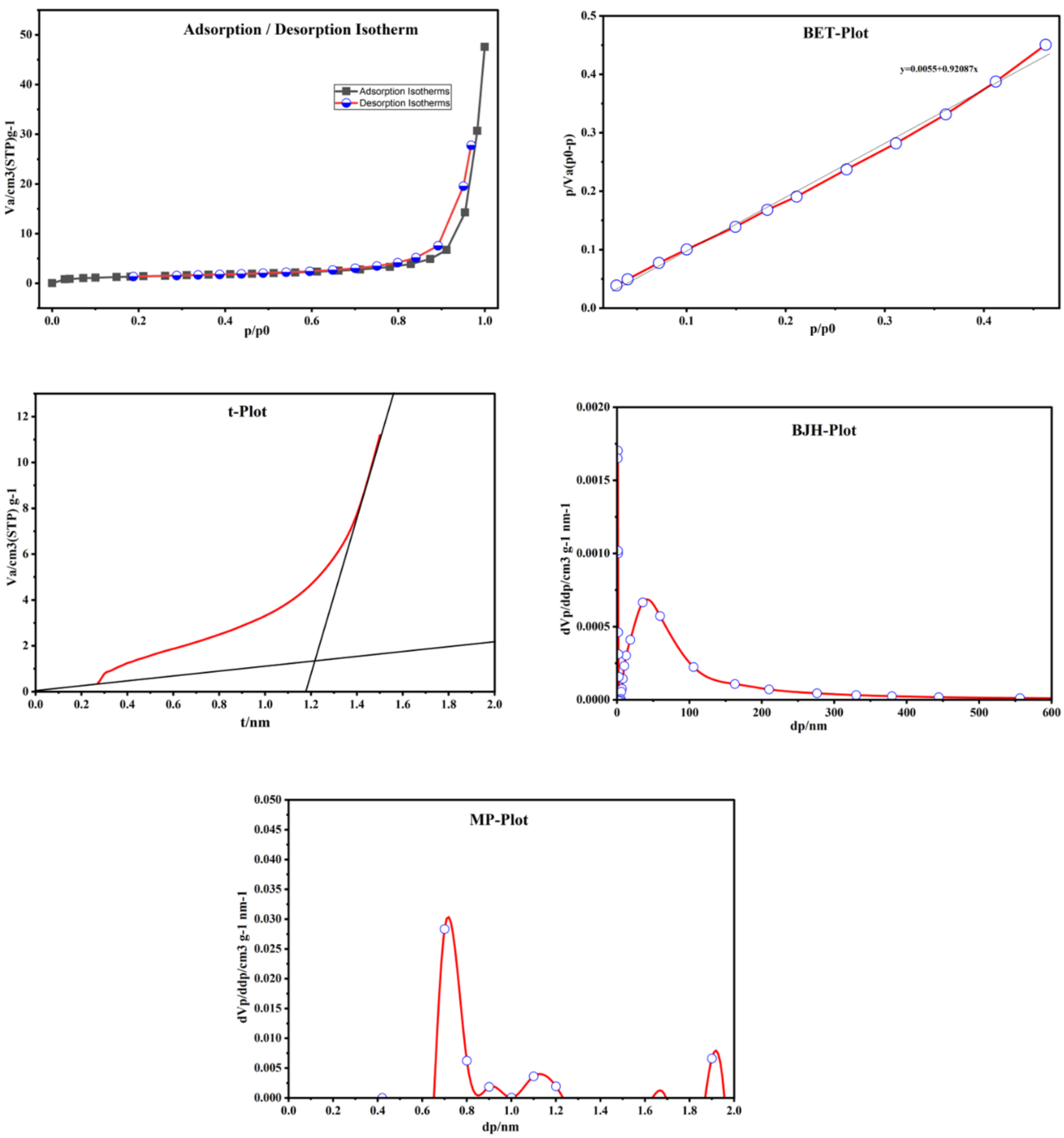

Figure S2. The BET analysis of Ag-ZnONPs

Table S1. List of materials used in the study

No.	Material/Reagent	Purity/Grade or Identification	Supplier/Source	Country
1	*Plantago ovata* seeds	Authenticated plant material	National Research Centre (NRC)	Egypt
2	Silver nitrate (AgNO₃)	≥99.9%	Sigma-Aldrich	USA
3	Copper nitrate	≥99%	Sigma-Aldrich	USA
4	Zinc nitrate	≥99%	Sigma-Aldrich	USA
5	Sodium hydroxide (NaOH)	≥98%	Merck	Germany
6	Ethanol	Analytical grade	Merck	Germany
7	Methanol	Analytical grade	Merck	Germany
8	Acetic acid	Analytical grade	Merck	Germany
9	Acetonitrile	HPLC grade	Merck	Germany
10	TRIzol reagent	Molecular-biology grade	Thermo Fisher Scientific	USA
11	Reverse-transcription kit	Molecular-biology grade	Thermo Fisher Scientific	USA
12	SYBR Green Master Mix	qPCR grade	Thermo Fisher Scientific	USA
13	Alexa Fluor® 488 Annexin V/Dead Cell Apoptosis Kit	Cat. No. V13241	Invitrogen/Thermo Fisher Scientific	USA
14	Vybrant™ DyeCycle™ Violet stain	Cat. No. V35003	Invitrogen/Thermo Fisher Scientific	USA
15	Bradford reagent	Protein-assay grade	Bio-Rad	USA
16	DPPH	Analytical grade	Sigma-Aldrich	USA
17	ABTS	Analytical grade	Sigma-Aldrich	USA
18	Potassium persulfate	Analytical grade	Sigma-Aldrich	USA
19	Ascorbic acid	Analytical grade	Sigma-Aldrich	USA
20	Mueller–Hinton agar	Microbiological grade	Oxoid	UK
21	Mueller–Hinton broth	Microbiological grade	Oxoid	UK
22	Tryptic soy broth	Microbiological grade	Oxoid	UK
23	Nutrient broth	Microbiological grade	Oxoid	UK
24	Crystal violet	Analytical grade	Supplier to be verified	—
25	Gentamicin	Antibacterial reference standard	Supplier to be verified	—
26	Caco-2 cell line	ATCC HTB-37	VACSERA	Egypt

No.	Material/Reagent	Purity/Grade or Identification	Supplier/Source	Country
27	MCF-7 cell line	ATCC HTB-22	VACSERA	Egypt
28	Vero cell line	ATCC CCL-81	VACSERA	Egypt
29	RPMI-1640 medium	Cell-culture grade	Gibco/Thermo Fisher Scientific	Germany
30	Dulbecco's Modified Eagle Medium (DMEM)	Cell-culture grade	Gibco/Thermo Fisher Scientific	Germany
31	Fetal bovine serum	Cell-culture grade	Gibco/Thermo Fisher Scientific	Germany
32	Penicillin–streptomycin–amphotericin B mixture	Cell-culture grade	Gibco/Thermo Fisher Scientific	Germany
33	Trypsin–EDTA	Cell-culture grade	Gibco/Thermo Fisher Scientific	Germany
34	Phosphate-buffered saline	Cell-culture grade	Gibco/Thermo Fisher Scientific	Germany
35	MTT reagent	Cell-culture grade	Supplier to be verified	—
36	Dimethyl sulfoxide (DMSO)	Cell-culture grade	Supplier to be verified	—
37	Doxorubicin	Anticancer reference standard	Supplier to be verified	—

Table S2 specific PCR primers were used for amplification of bacterial virulence genes

FW-FnbA	AGCGGTAACCAGTCATTCGAG	50	52	this study
RW-FnbA	TTGGCGGCGTTGTATCTTCT	50	50	this study
FW-Cna	AAGGTGAACAGGTGGGTCAA	59	50	this study
FW-Cna	CACTACTTGTTCCCGCTTCA	57	50	this study
FW-algD	TCAACAACGTGGTGATCCCG	60.5	55	this study
RW-algD	AGCTCGCGGTAGATTTCCTC	60	55	this study
FW-lasB	GAACGACTTCCTGATCGGCT	59	50	this study
RW-lasB	AACAGGTAGAACGCACGGTT	59	55	this study
FW-RecA	GATATGCACGTTGGGTTGGC	60	55	this study
RwW-RecA	TCGTCACCTTCCTTCACTGC	60	55	this study

Table S3. Primer sequences used for qRT-PCR analysis

Gene	Primer	Sequence (5′–3′)	Ref.
Caspase-3	Forward	GTGGAGGCCGACTTCTTGTA	[1]
	Reverse	TGTCGGCATACTGTTTCAGC	
BAX	Forward	AGACACCTGAGCTGACCTTGGAG	[2]
	Reverse	GTTGAAGTTGCCATCAGCAAACA	
Bcl-2	Forward	TGAACCGGCATCTGCACAC	[2]
	Reverse	CGTCTTCAGAGACAGCCAGGAG	

Table S4. Batch-to-batch reproducibility of independently prepared *Plantago ovata* aqueous extracts.

**Parameter**	**E1**	**E2**	**E3**	**Mean ± SD**	**CV (%)**
pH	6.20	6.60	6.63	6.48 ± 0.24	3.71
Dry solids (mg/mL)	7.56	8.65	8.78	8.33 ± 0.67	8.04
Extraction yield (%)	15.12	17.30	17.56	16.66 ± 1.34	8.04
